# *In vivo* monitoring of the recruitment and activation of AP-1 by Arf1

**DOI:** 10.1038/s41598-017-07493-1

**Published:** 2017-08-02

**Authors:** Etienne Sauvageau, Peter J. McCormick, Stephane Lefrancois

**Affiliations:** 1 Centre INRS-Institut Armand-Frappier, INRS, Laval, Canada H7V 1B7; 2 Faculty of Health and Medical Sciences, School of Veterinary Medicine, University of Surrey, Guildford, GU27XH UK; 3 Department of Anatomy and Cell Biology, McGill University, Montreal, Canada H3A 2B2

## Abstract

AP-1 is a clathrin adaptor recruited to the trans-Golgi Network where it can interact with specific signals found in the cytosolic tail of cargo proteins to incorporate them into clathrin-coated vesicles for trafficking. The small G protein Arf1 regulates the spatiotemporal recruitment of AP-1 and also drives a conformational change favoring an interaction with cargo proteins. A recent crystal structure and *in vitro* experiments highlighted potential residues mediating the AP-1/Arf1 interaction and the unlocking of the complex. We have used bioluminescence resonance energy transfer (BRET) to study the Arf1/AP-1 interaction and AP-1 conformational changes *in vivo*. We identified novel residues required for this interaction in addition to those predicted in the crystal structure. We also studied the conformational changes in AP-1 driven by Arf1 in live cells and found that opening of the complex is prerequisite for oligomerization. Using Arf1 knockout cells generated by CRISPR/Cas9, we demonstrated that residue 172 in Arf1 is necessary for AP-1 activation and is required for the efficient sorting of the lysosomal protein prosaposin. We have used BRET to study the *in vivo* activation of AP-1. The advantages of BRET include expressing full-length proteins in their native environment that have been fully post-translationally modified.

## Introduction

The formation of clathrin-coated vesicles (CCVs) plays an important role in the intracellular transport of integral membrane and intraluminal proteins from one membrane compartment of the cell to another. Adaptor protein complexes perform dual roles in binding specific peptide motifs on membrane-associated cargo proteins and recruit clathrin to drive vesicle formation^1^.

The adaptor protein complex 1 (AP-1) is a heterotetrameric complex composed of two large subunits (β1 and γ), a medium subunit (µ1) and a small subunit (σ1) involved in traffic between the trans-Golgi network (TGN) and endosomes^2, 3^. Structurally, AP-1 consists of a “core” domain made of the N-terminal portions of the β1 and γ subunits plus the entire µ1 and σ1 subunits. Outside of the core domain, the C-terminal part of β1 and γ form two “appendage” domains involved in the interaction with clathrin and other accessory proteins^3^. The core domain recruits the complex to membranes and recognizes sorting sequences in the cytosolic tail of cargo proteins such as tyrosine (YXXØ, where X is any amino acids and Ø is a bulky hydrophobic amino acid) or dileucine ([D/E]XXXL[L/I])-type signals^4–6^. Besides interaction with cargo proteins, binding of the γ subunit to phosphatidylinositol-4-phosphate (PI(4)P) also facilitates AP-1 recruitment to membranes^7^. However, the main factor targeting AP-1 to the membrane of endosomes and the TGN are members of the ADP ribosylation factor (Arf) family of small GTPases. Arf1, for example, recruits AP-1 to TGN membranes, followed by the binding of clathrin to the adaptors and the formation of CCVs^8, 9^. Whereas the GDP bound form of Arf1 is cytosolic, the exchange of GDP for GTP leads to conformational changes in Arf1 exposing a myristoyl group and an associated N-terminal amphipathic helix, critical for Arf1 membrane binding^10, 11^. These conformational changes extend to the switch I-II domains of Arf1, allowing it to interact with various effectors such as AP-1.

The crystal structure of the AP-1 core domain with a GTP-bound N-terminal truncated form of Arf1, revealed two interaction interfaces between the switch I-II domains of Arf1 and the β1 and γ subunits^12^. *In vitro* binding experiments suggest that these interfaces are necessary for high affinity binding of AP-1 to Arf1^12^. Another striking feature of this crystal structure seems to suggest that Arf1 drives a conformational change in the AP-1 complex from a “locked” to an “open” conformation able to interact with tyrosine or dileucine-based signals of cargo proteins^12, 13^. Based on *in vitro* experiments, a third interaction interface between the C-terminal back-side of Arf1 and the γ subunit of AP-1, would not be necessary for the interaction between these two proteins, but would participate in the allosteric activation of AP-1^12^. So far, much of what is known regarding AP-1 recruitment and activation is based on *in vitro* experiments using truncated forms of the proteins. However, the next step toward a more complete understanding of these mechanisms will require the study of full-length forms of AP-1 and Arf1 in their natural cellular environment.

In recent years, resonance energy transfer (RET)-based techniques like fluorescence resonance energy transfer (FRET) and bioluminescence resonance energy transfer (BRET) have increasingly been used to study protein-protein interaction^14, 15^, intra or inter-molecular conformational changes^16–19^ and ligand-binding^20^ in living cells, in real time and with proteins expressed in their natural environment. RET is a phenomenon occurring when the emission spectrum of a donor chromophore overlaps with the excitation spectrum of an acceptor chromophore allowing for the non-radiative transfer of energy from the donor, following its excitation, to the acceptor molecule. The RET efficacy is inversely proportional to the sixth power of the distance between the donor and acceptor and will not occur if the two chromophores are separated by more than ~10 nm^21^. As such, the occurrence of RET between chromophores attached to two proteins can be interpreted as a direct interaction between these proteins or their presence in a multi-protein complex. In BRET, the donor chromophore is the bioluminescent enzyme *Renilla* luciferase (RlucII), whereas in FRET it is a photoexcitable chromophore. Since RlucII is excited by the addition of an enzyme substrate instead of an external light beam, as is the case for FRET, the main advantage of BRET over FRET is the absence of background emission coming from non-specific cell excitation.

Therefore, we sought to study the interaction between Arf1 and AP-1 and the conformational changes in the AP-1 complex in living cells using BRET^2^ technology. We found that BRET^2^ can detect the interaction between Arf1 and AP-1 and the decreased affinity caused by mutations in Arf1 or AP-1 subunits. BRET^2^ was also successfully used to detect the conformational changes associated with the activation of AP-1 and the role Arf1 plays in the unlocking of the complex. Finally, we found that Arf1 increases AP-1 oligomerization by recruiting it to membranes and changing its conformation.

## Results

### Detection of the Arf1/AP-1 interaction using BRET

**Figure 1:**
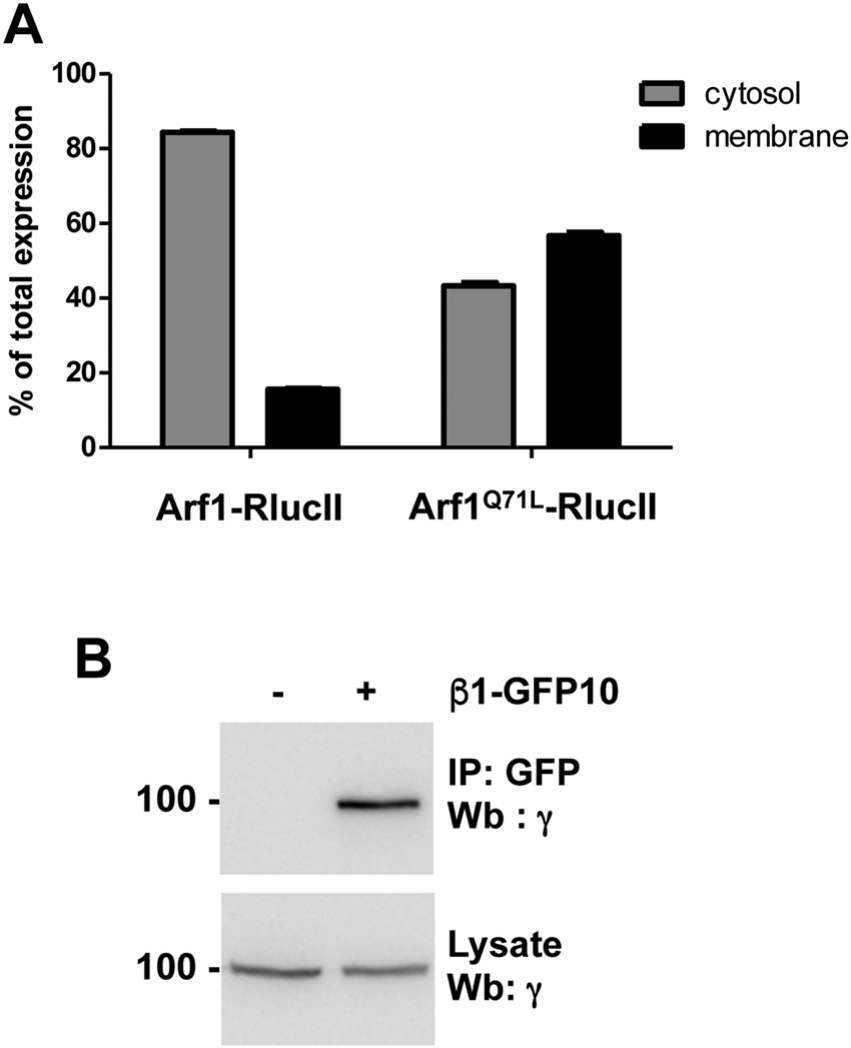
Functionality of the RlucII and GFP10 constructs used for BRET. (**A**) Arf1 recruitment to membranes was measured in HEK293T cells transfected with either Arf1-RlucII or Arf1^Q71L^-RlucII. Cytosol (grey bars) and membrane compartments (black bars) were separated by cell fractionation and the luminescence was measured in each fraction. Results are expressed as a % of total luminescence. (**B**) Co-immunoprecipitation of β1-GFP10 with endogenous γ was detected in HEK293T cells transfected with β1-GFP10. β1-GFP10 was immunoprecipitated with an antibody against GFP and endogenous γ was detected by Western blot (Wb) using an anti-γ antibody. Western blot images were cropped for space considerations.

In order to detect the formation of a complex between Arf1 and AP-1 in living cells, Arf1 was fused at its C-terminus to the energy donor *Renilla* luciferase II (Arf1-RlucII) and the β1 and γ subunits of AP-1 were fused at their C-terminus to the blue-shifted green fluorescent protein 10 (β1-GFP10 or γ-GFP10) energy acceptor. Although GFP-tagged version of Arf1^22, 23^ and β1^12^ have been described before, we first sought to determine if the presence of a larger tag like RlucII or GFP10 could affect the function of either Arf1 or β1. As seen in Fig. 1A, a membrane isolation assay performed in HEK293T cells transfected with Arf1-RlucII confirms that Arf1 retains its ability to bind membranes when fused to RlucII. Moreover, a GTP-locked mutant of Arf1 (Arf1^Q71L^-RlucII) displays increased membrane association (Fig. 1A), suggesting that the presence of RlucII at the C-terminus of Arf1 does not interfere with its activation. Nevertheless, it was previously shown that the presence of a c-terminal tag on Arf1 (GFP or HA) can affect its GTP exchange and hydrolysis^24^ and we cannot exclude the possibility that the presence of the RlucII tag might influence Arf1 function. We then sought to determine if the presence of GFP10 at the C-terminus of β1 could interfere with its ability to interact with other AP-1 subunits. For this purpose, β1-GFP10 was transfected in HEK293T cells and immunoprecipitated using an antibody against GFP. Western blot analysis using an antibody against the γ subunit revealed that β1-GFP10 interacts with endogenously expressed γ (Fig. 1B), suggesting that it is integrated into AP-1 complexes.

**Figure 2:**
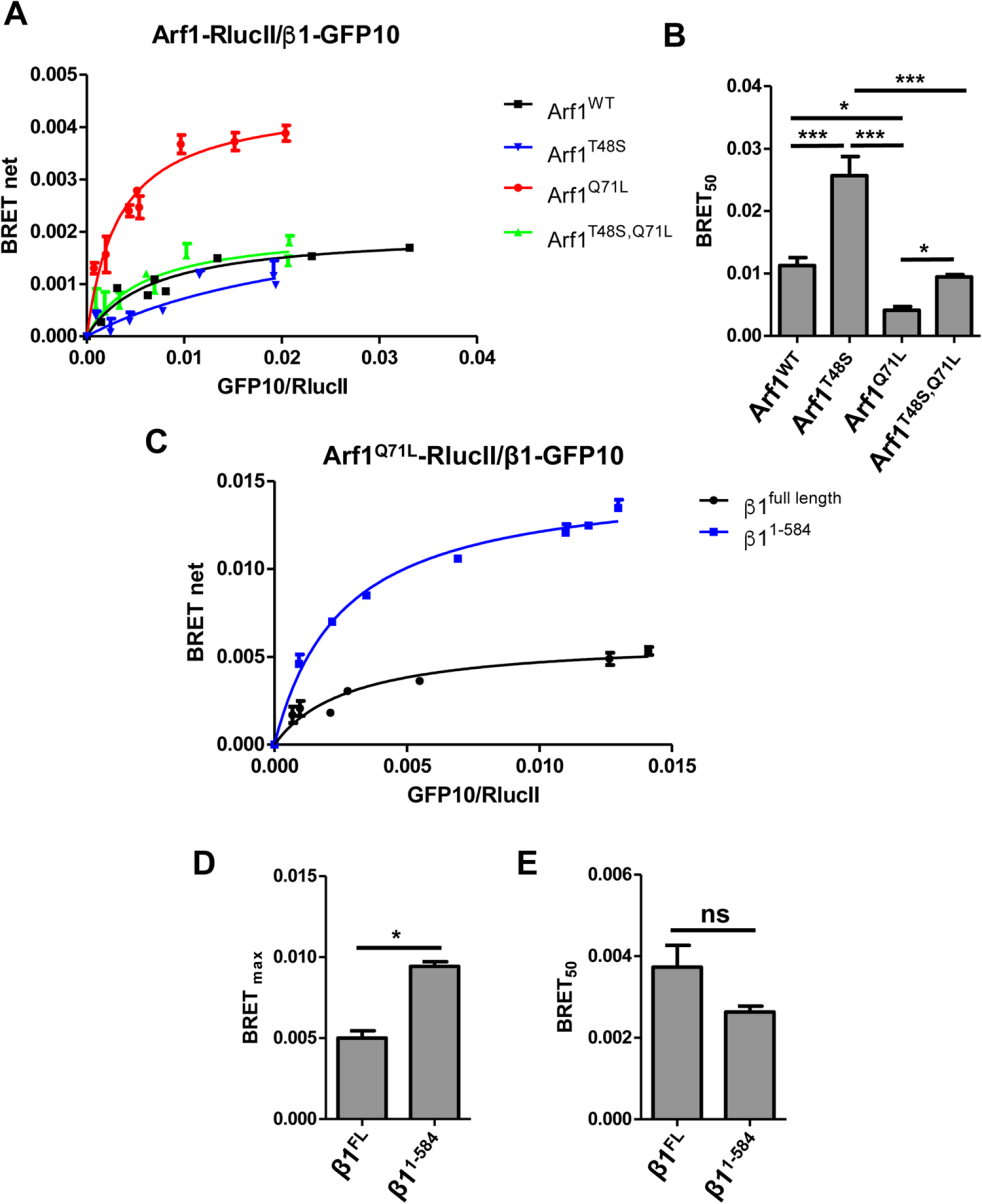
Detection of the interaction between Arf1 and the AP-1 subunit β1 in HEK293T cells using BRET. (**A**) HEK293T cells were transfected with a fixed concentration of Arf1-RlucII (black curve), Arf1^Q71L^-RlucII (red curve), Arf1^T48S^-RlucII (blue curve) or Arf1^T48S,Q71L^-RlucII (green curve) and increasing concentrations of β1-GFP10. Fluorescence and BRET^2^ were measured in parallel. BRET_net_ levels are plotted as a function of the ratio of fluorescence over luminescence (GFP10/RlucII). Data points are mean ± SEM of triplicates. (**B**) BRET_50_ for each Arf1 construct are shown as mean ± SEM of 4 independent experiments analyzed by ANOVA followed by Tukey’s post-hoc test. *P < 0.05; ***P < 0.001; ns, not significant. (**C**) Arf1^Q71L^-RlucII was co-transfected with increasing quantities of full-length β1-GFP10 (black curve) or truncated β1^1–584^-GFP10 (blue curve) and BRET^2^ signals were measured. Data points are mean ± SEM of triplicates. (**D**) BRET_max_ and (**E**) BRET_50_ are represented as the mean ± SEM of three independent experiments analyzed by paired student *t*-test to assess statistical significance. *P < 0.05; ns, not significant.

We next detected the association between Arf1 and AP-1 in living cells by generating BRET titration curves in which a fixed amount of the donor Arf1-RlucII (WT or mutant) was co-transfected in HEK293T cells with increasing quantities of the acceptor β1-GFP10. As seen in Fig. 2A (black line), the accumulation of β1-GFP10 gradually increases the BRET signal with wild-type Arf1-RlucII ultimately reaching a plateau, suggesting that the BRET signal is coming from a specific interaction and not random collision^25^. The highly conserved threonine 48 residue of Arf1, involved in the GDP to GTP exchange^26^, was mutated to serine to decrease the activation of Arf1. This mutation did not alter the expression level of Arf1 compared to wild-type (Figure S1), but it lead to a decrease of BRET with β1-GFP10 and a linearization of the titration curve (Fig. 2A, blue line). As seen in Fig. 2A (red line), the constitutively active Arf1^Q71L^-RlucII displays higher BRET signal with β1-GFP10 and the BRET_50_, the GFP10/RlucII ratio giving 50% of the BRET_max_ signal, is significantly lower than ARF1^WT^, suggesting a higher propensity to interact with AP-1 which is consistent with *in vitro* observations (Fig. 2B). Simultaneously mutating T48S and Q71L (Arf1^T48S,Q71L^-RlucII) brought the BRET_max_ (Fig. 2A) and BRET_50_ (Fig. 2B) to similar levels as for wild-type Arf1. The relatively low BRET signals observed between Arf1 and AP-1 are probably explained by the distance between the RlucII and GFP10 tags as Arf1 sits very close to the membrane whereas the C-terminal extension of β1 is thought to be floating in the cytosol away from the membrane. We generated BRET titration curves with full length β1 and β1^1–584^ with Arf1^Q71L^-RlucII (Fig. 2C). Indeed, removing the cytosolic ear of β1 and placing the GFP10 tag just outside the trunk of AP-1 (β1^1–584^) significantly increased the BRET_max_ (Fig. 2D) with Arf1^Q71L^ whereas the BRET_50_ (Fig. 2E) was unaffected, suggesting that the C-terminal extension of β1 does not influence the interaction with Arf1. Truncation of β1 did not affect its expression compared to wild-type β1 (Figure S2A). It has been suggested that the hyperbolic form of a BRET titration curve is not sufficient to conclude that the BRET signal is coming from a specific interaction^27, 28^. An alternative approach is to perform the BRET titration curves at different levels of donor expression and express the BRET ratio as a function of acceptor expression. In the case of non-specific interaction, the BRET ratio should be independent of donor levels, whereas for a specific interaction the BRET ratio should decrease with increasing levels of donor expression at similar levels of acceptor fluorescence^28^. As seen in Figure S3, the BRET ratios between Arf1^WT^-RlucII (Figure S3A) or Arf1^Q71L^-RlucII (Figure S3B) and β1-GFP10 decrease with increasing donor concentration confirming that the BRET signal observed between Arf1 and β1 is coming from a specific interaction.

### Reduced interaction between Arf1 and AP-1 mutants is detected by BRET *in vivo*

**Figure 3:**
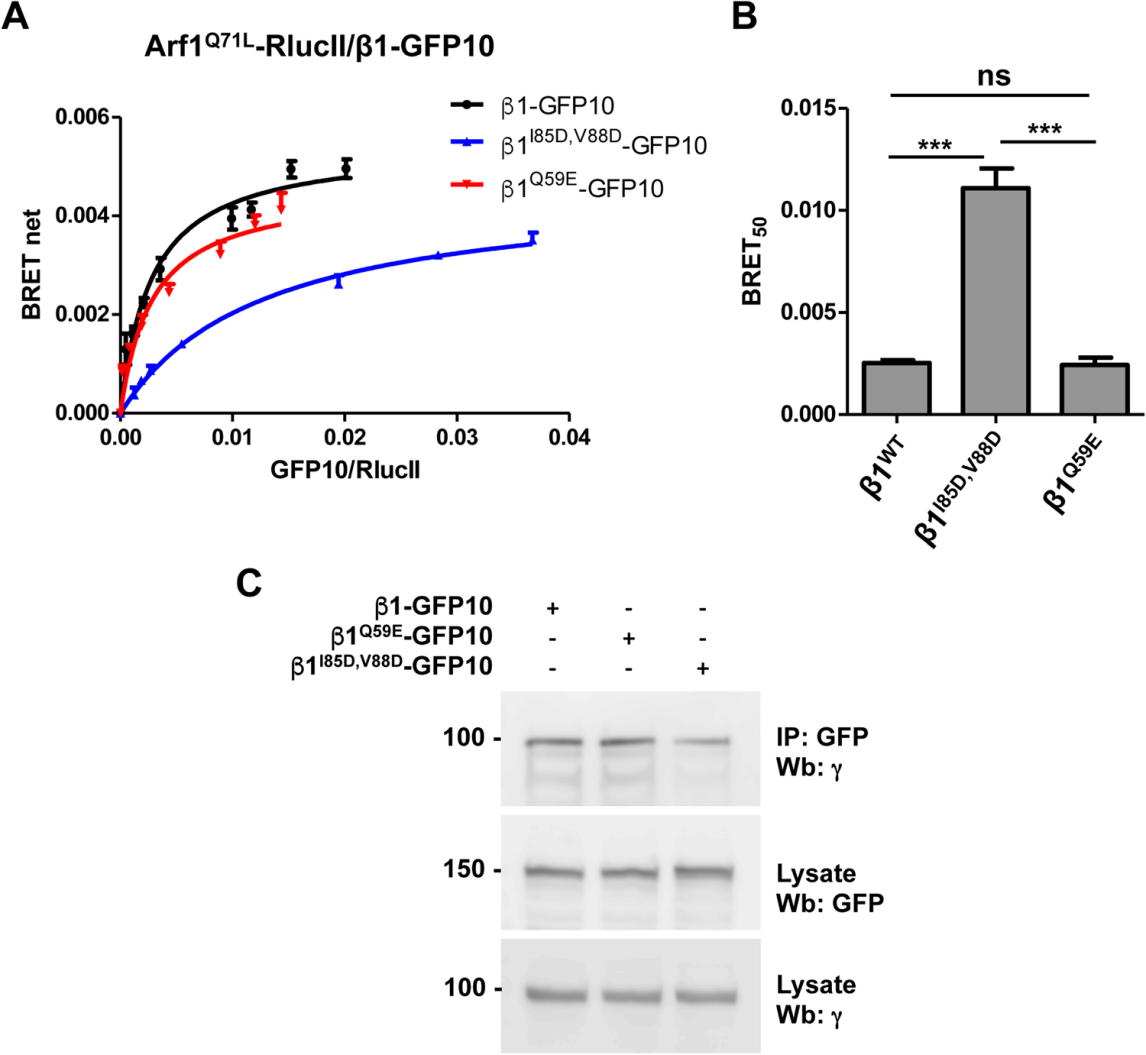
Mutations in β1 leads to decreased interaction between Arf1 and AP-1 as detected by BRET titration experiments in living cells. (**A**) The effect of mutating the β1 subunit on binding was tested in HEK293T cells transfected with a low and constant amount of Arf1^Q71L^-RlucII and increasing concentrations of β1-GFP10 (black curve), β1^I85D,V88D^-GFP10 (blue curve) or β1^Q59E^-GFP10 (red curve). (**B**) BRET_50_ for each β1 construct as represented as the mean ± SEM of 4 independent experiments analyzed by ANOVA followed by Tukey’s post-hoc test. ***P < 0.001; ns, not significant. (**C**) β1-GFP10 or the indicated mutant were immunoprecipitated using an anti-GFP antibody and the amount of endogenous γ co-immunoprecipitated was detected by western blot using an anti-γ antibody. Lysate represents 10% of the input. Western blot images were cropped for space considerations.

**Figure 4:**
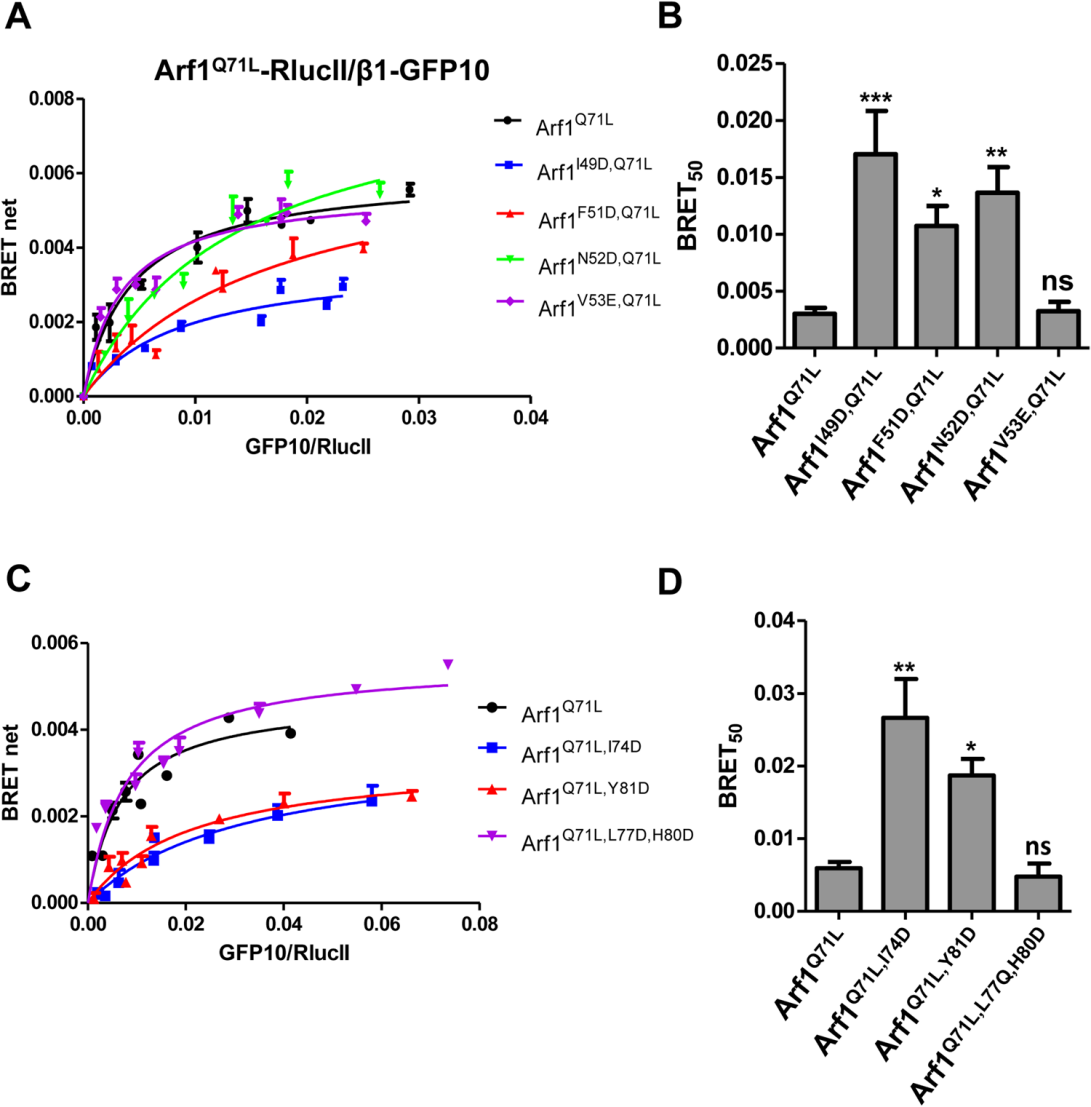
Reduced interaction between AP-1 and Arf1 mutants detected by BRET. Arf1^Q71L^-RlucII or various switch 1 (**A**) or switch 2 (**C**) mutated forms of Arf1 were transfected at a low and constant amount along with increasing quantities of β1-GFP10 into HEK293T cells. The BRET^2^ signals were measured. BRET_50_ for each switch 1 (**B**) or switch 2 (**D**) mutated forms of Arf11 are shown as the mean ± SEM of 5 independent experiments analyzed by ANOVA followed by Dunnett’s post-hoc test. *P < 0.05; **P < 0.01; ***P < 0.001; ns, not significant.

The crystallization of a truncated form of AP-1 with a truncated form of Arf1 lacking its N-terminus conjugated to GTP revealed a potential interaction interface between the β1 subunit of AP-1 and the switch 1 and 2 regions of Arf1. Mutation of some residues in that interface led to reduced binding *in vitro* ^12^. We first wanted to confirm the importance of these residues for the interaction between full-length AP-1 and Arf1 *in vivo* using BRET. Arf1^Q71L^-RlucII was transfected into HEK293T cells with increasing concentrations of wild-type β1-GFP10 or β1^I85,V88D^-GFP10, at similar levels of expression (Figure S2B), to perform BRET titration experiments. As shown in Fig. 3, the BRET titration curve for the mutated form of β1 (Fig. 3A, blue line) is shifted to the right leading to a higher BRET_50_ (Fig. 3B) confirming the importance of these residues for the interaction between Arf1 and AP-1 *in vivo*. In contrast, the mutation of glutamine 59 (β1^Q59E^-GFP10), another β1 residue suggested by the crystal structure to make contact with Arf1, had no effect on the BRET_50_ (Fig. 3B) indicating that mutating this residue alone is not sufficient to lead to a significant loss of binding. To ensure that the changes in the interaction of β1^I85,V88D^-GFP10 with Arf1 we observed in BRET were not a result of the mutant β1 not binding the other subunits of the complex and therefore not being incorporated into the complex, we performed a co-immunoprecipitation experiment (Fig. 3C). Wild-type β1 and the two mutants were able to interact with endogenous γ, suggesting that the three β1 proteins were integrated into AP-1 complexes. BRET titration experiments between β1-GFP10 and wild-type or mutated forms of Arf1-RlucII were next performed to test the role of the switch 1 domain of Arf1 in the interaction with AP-1 *in vivo* (Fig. 4A). As seen in Fig. 4B, Arf1^I49D,Q71L^ shows reduced interaction with β1-GFP10 (larger BRET_50_ value) compared to Arf1^Q71L^, confirming results previously obtained *in vitro*. However, mutation of valine 53 (Arf1^V53E,Q71L^), which significantly decreased the interaction *in vitro*, had no effect on the BRET between Arf1 and β1 suggesting that this residue does not strongly contribute to the association *in vivo* (Fig. 4B). Two novel mutations in the switch 1 region of Arf1 (F51D and N52D) also led to a rightward shift of the BRET titration curve (Fig. 4A, red and purple lines) and a corresponding increase in their BRET_50_ (Fig. 4B) confirming the role of this domain in the AP-1/Arf1 association. Similar experiments were also done with mutations in the switch 2 region of Arf1 (Fig. 4C). Interestingly, Arf1^Q71L,L77D,H80D^, a mutant shown to lose all interaction with AP-1 *in vitro*, displayed similar BRET_50_ to Arf1^Q71L^ *in vivo*, suggesting a minimal role of these residues in the AP-1/Arf1 interaction *in vivo* (Fig. 4D). However, two novel mutations (Y74D and Y81D) showed a significant increase in BRET_50_ compare to wild-type (Fig. 4D), confirming that the switch 2 domain is also involved in the recruitment of AP-1 to Arf1. All Arf1 mutants used in this study maintained co-localization with the Golgi marker giantin (Figures S4 and S5) suggesting that any loss in interaction was not due to mis-localization of Arf1.

**Figure 5:**
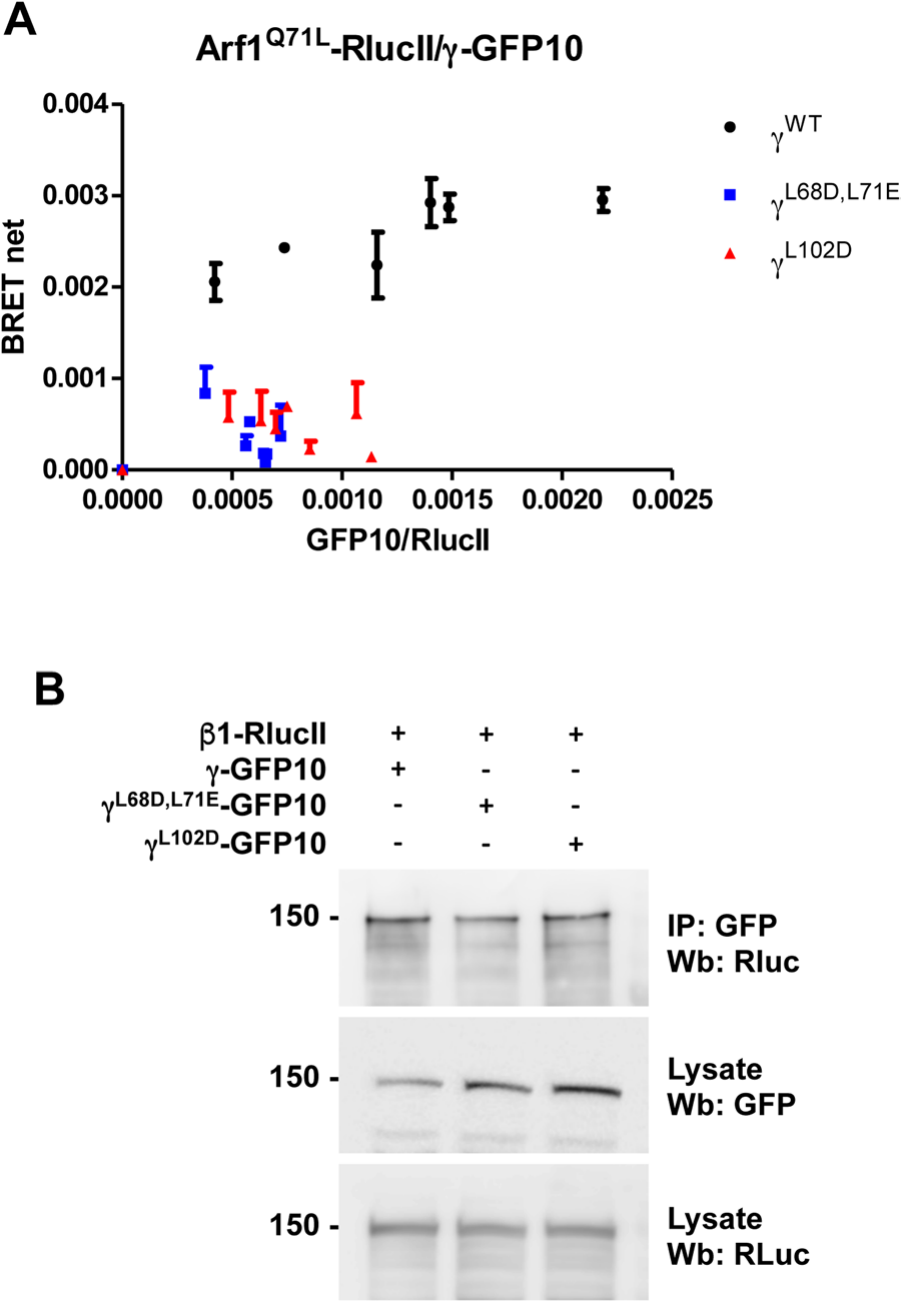
Effect of mutating the γ subunit on the interaction between AP-1 and Arf1. (**A**) This interaction was tested in HEK293T cells transfected with a constant amount of Arf1^Q71L^-RlucII and increasing concentrations of γ-GFP10 (black dots), γ ^L68D,L71E^-GFP10 (blue dots) or γ^L102D^-GFP10 (red dots). BRET^2^ signals were measured. (**B**) HEK293T cells were co-transfected with wild-type (WT) γ-GFP10 or the indicated mutants and β1-RLucII. γ-GFP10 was immunoprecipitated using an anti-GFP antibody and the amount of β1-RlucII co-immunoprecipitated was detected by Western blot (Wb) using an anti-RLuc antibody. Western blot images were cropped for space considerations.

Though not visible in the crystal structure, a second binding interface between the switch 1/2 region and the N-terminus of the γ subunit has also been proposed to be involved in AP-1 recruitment to Arf1^12^. We sought to determine if residues in γ essential for the binding to Arf1 *in vitro*, are also important *in vivo* by performing BRET titration experiments. Wild-type γ efficiently binds to Arf1 as shown by the saturated curve (Fig. 5A, black line). The two γ mutants (γ^L68D,L71D^ and γ^L102D^) display a significant decrease in BRET signal compare to wild-type γ, at similar levels of expression (Figure S2C), suggesting that this interface also plays a role in the AP-1/Arf1 association *in vivo* (Fig. 5A). The absence of saturation for γ^L68D,L71D^ and γ^L102D^ (Fig. 5A, blue and red points) suggest these γ mutants have completely lost their ability to interact with Arf1 in cells. The change in the interaction with Arf1 was not due to the mutant γ subunits not integrating into the AP-1 complex as wild-type and mutant γ were able to co-immunoprecipitate with β1-RlucII (Fig. 5B).

### Monitoring AP-1 conformational changes by BRET

**Figure 6:**
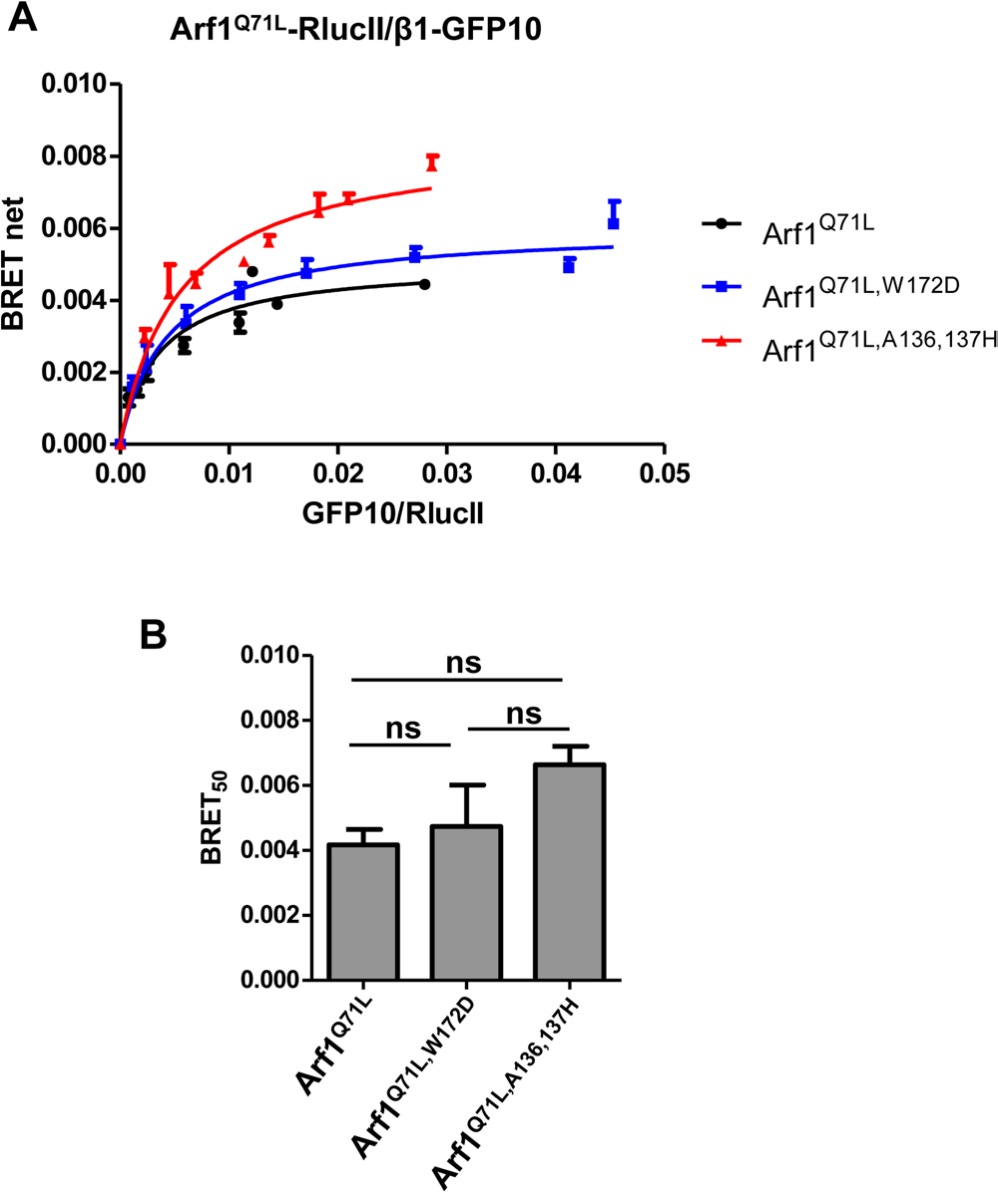
The back-side region of Arf1 does not contribute to AP-1 binding *in vivo*. (**A**) BRET titration curves were generated by transfecting HEK293T cells with Arf1^Q71L^-RlucII (black curve), Arf1^Q71L,W172D^-RlucII (blue curve) or Arf1^Q71L,A136,137H^-RlucII and increasing quantities of β1-GFP10 and BRET signals were measured. (**B**) BRET_50_ for each Arf1 construct are shown as the mean ± SEM of 3 independent experiments analyzed by ANOVA followed by Tukey’s post-hoc test. ns, not significant.

The crystal structure of AP-1 with Arf1-GTP revealed another binding interface between the back-side of Arf1 and the γ subunit of AP-1. Based on *in vitro* experiments, this interface would not be necessary for AP-1 recruitment to Arf1, but would participate in the activation of AP-1 by unlocking its conformation and allowing the C-terminal domain of µ1 to interact with membrane cargo^12^. We first tested the role of this interface *in vivo* by generating BRET titration curves between wild-type Arf1-RlucII or various Arf1 mutants and β1-GFP10 (Fig. 6A). Mutations in this region of Arf1 (W172D or A136, 137H) resulted in no significant changes in BRET_50_ suggesting that this interface is not involved in AP-1 binding to Arf1 (Fig. 6B). An increase in BRET_max_ observed with Arf1^Q71L,A136,137H^-RlucII suggests that these mutations alter the conformation of Arf1 but not its ability to bind AP-1 (Fig. 6A, red line).

**Figure 7:**
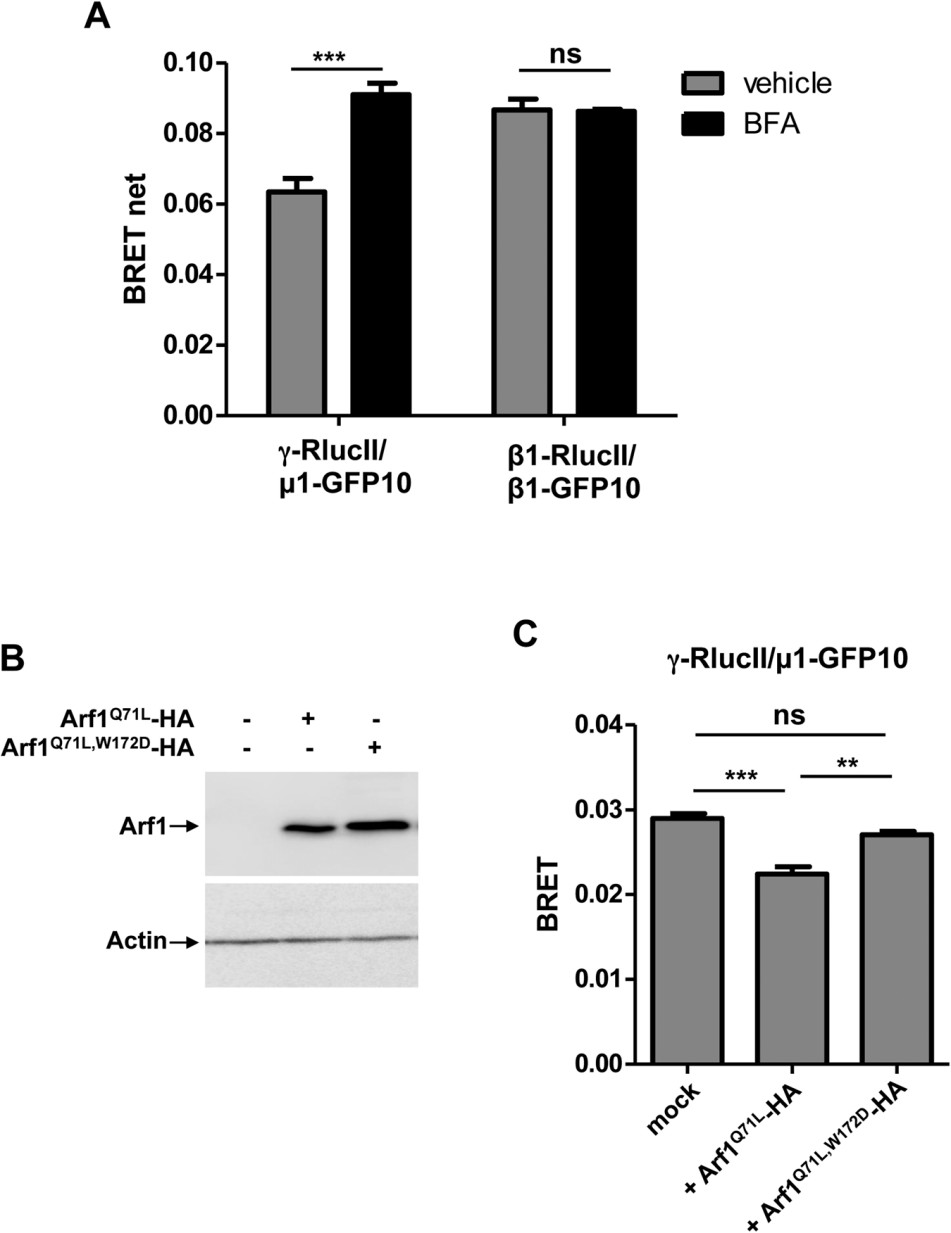
The back-side region of Arf1 participates in the allosteric activation of AP-1 *in vivo*. (**A**) HEK293T cells transfected with γ-RlucII/µ1-GFP10 or β1-RlucII/β1-GFP10 were treated with vehicle (grey bars) or Brefeldin A (BFA, black bars) and BRET signals were measured. Data are represented as the mean ± SEM of three independent experiments analyzed by paired student *t*-test to assess statistical significance. ***P < 0.001; ns, not significant.(**B**) γ-RlucII and µ1-GFP10 were transfected in HEK293T cells with or without Arf1^Q71L^-HA or Arf1^Q71L,W172D^-HA and BRET signals were measured. BRET signals shown are observed at similar levels of fluorescence/luminescence ratios. Data are shown as the mean ± SEM of six independent experiments analyzed by one-way ANOVA followed by Tukey’s post-hoc test to assess statistical significance. **P < 0.01; ***P < 0.001; ns, not significant. (**C**) The level of expression of Arf1^Q71L^-HA and Arf1^Q71L,W172D^-HA were determined by Western blot using an anti-HA antibody.

**Figure 8:**
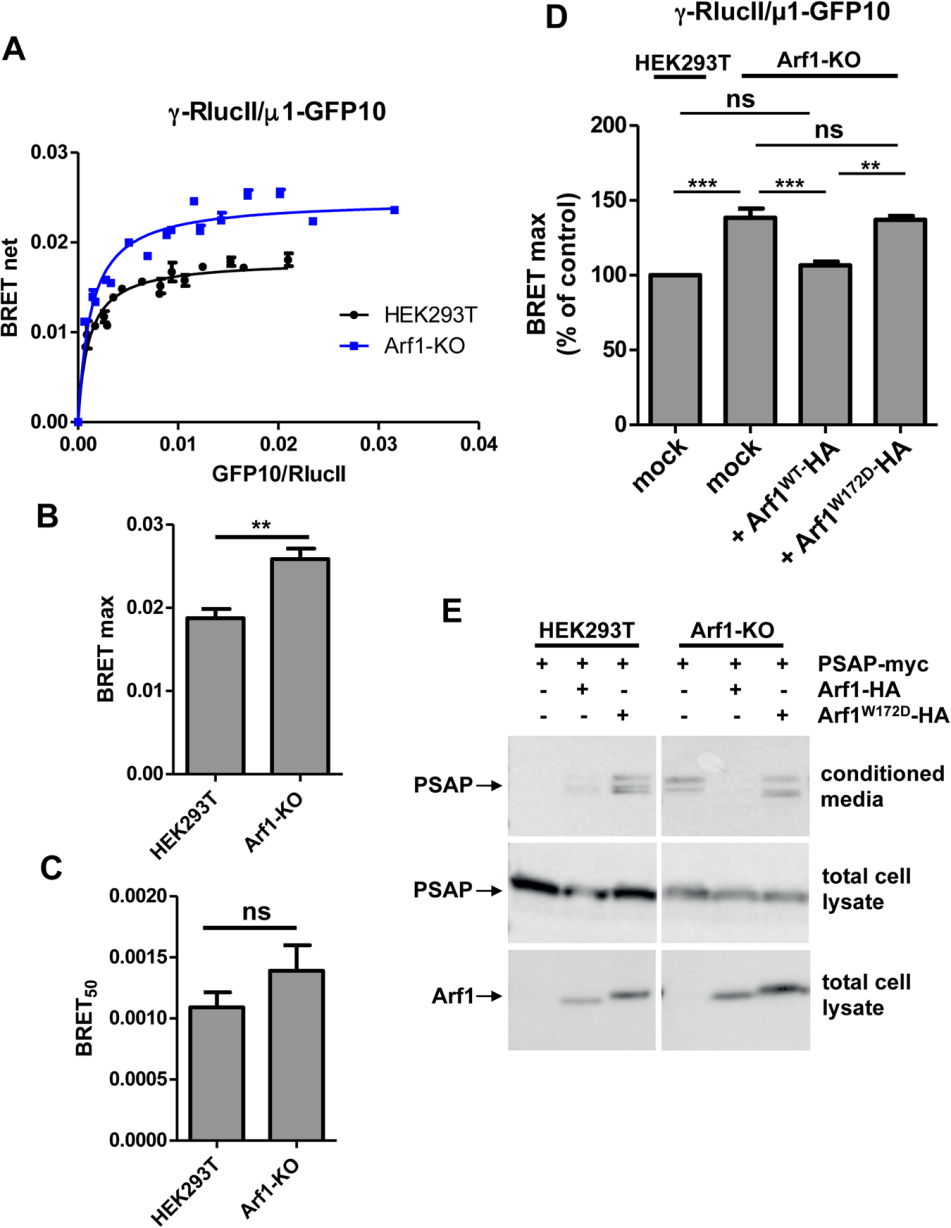
Rescue experiments in Arf1-KO cells confirms the allosteric function of Arf1 back-side (**A**). A constant amount of γ-RlucII along with increasing quantities of µ1-GFP10 were transfected in HEK293T (black curve) or Arf-KO (blue curve) cells and BRET signals were measured. Data points are shown as mean ± SEM of triplicates. BRET_50_ (**B**) and BRET_max_ (**C**) are represented as mean ± SEM of 4 independent experiments analyzed by ANOVA followed by Tukey’s post-hoc test. **P < 0.01; ns, non significant.(**D**) HEK293T or Arf1-KO cells were transfected with γ-RlucII, µ1-GFP10 and Arf1-HA or Arf1^W172D^-HA and BRET signals were measured. BRET signals shown are observed at similar level of fluorescence/luminescence ratios. Data is represented as mean ± SEM of 4 independent experiments and their statistical significance were assessed by ANOVA followed with Tukey’s post-hoc test. **P < 0.01; ***P < 0.001; ns, non significant.(**E**) HEK293T or Arf1-KO cells were transfected with prosaposin-myc (PSAP-myc) with or without Arf1-HA or Arf1^W172D^-HA. The amount of PSAP-myc secreted in the media was determined by western blot using an anti-myc antibody. Western blot images were cropped for space considerations.

We next sought to monitor AP-1 conformational changes by tagging the γ subunit to RlucII (γ-RlucII) and the µ1 subunit to GFP10 (µ1-GFP10) and co-transfecting them into HEK293T cells. First, cells were treated with Brefeldin A (BFA), an Arf1 GEF inhibitor that leads to the inactivation of AP-1^29^ or vehicle for the indicated times and the BRET between γ-RlucII and µ1-GFP10 was measured. BFA treatment leads to an increase in BRET between the two AP-1 subunits (Fig. 7A) suggesting that the C-terminus of γ and µ1 are closer and in the “locked” conformation. It has been shown that AP-1 can oligomerize^30^ and to decrease the possibility that the BRET increase observed reflects a change in the oligomerization status of AP-1 rather than a change in conformation, these experiments were performed at BRET_max_ where moderate changes in the oligomerization of AP-1 should have minimal effects on the BRET between γ-RlucII and µ1-GFP10. Moreover, BFA treatment should lead to a dissociation of AP-1 from the membrane and a decrease in the oligomerization of AP-1 that could not explain the increase in BRET between γ-RlucII and µ1-GFP10. Nevertheless, we also performed the BFA treatment experiment on cells transfected with β1-RlucII and β1-GFP10 where the BRET signal can only come from different AP-1 complexes. When performing the assay at BRET_max_, we saw no changes in BRET (Fig. 7A) between β1-RlucII and β1-GFP10, confirming that the increase in BRET between γ and µ1 comes from a conformational change of the complex and not from oligomerization. If this is true, increasing the proportion of active AP-1 in cells should lead to a decrease in BRET between γ-RlucII and µ1-GFP10. In order to test this, the two AP-1 subunits were co-transfected with a constitutively active form of Arf1 (Arf1^Q71L^-HA) or a mutant form (Arf1^Q71L,W172D^-HA) that was shown *in vitro* not to open the complex (Fig. 7B). As expected, expressing Arf1^Q71L^-HA resulted in a decrease in BRET between γ-RlucII and µ1-GFP10 (Fig. 7C). Interestingly, overexpression of Arf1^Q71L,W172D^-HA only slightly decreased the BRET between γ-RlucII and µ1-GFP10 (Fig. 7C) suggesting that even though this mutant binds normally to AP-1 (Fig. 6B), it is less efficient at driving AP-1 unlocking. We sought to confirm this result by performing rescue experiments in a CRISPR/Cas9 generated Arf1 knockout (Arf1-KO) cell line (Figure S6). The morphology, survival and growth rate of these cells is comparable to wild-type HEK293T cells. BRET titration curves between γ-RlucII and µ1-GFP10 were first performed in HEK293T or Arf1-KO cells (Fig. 8A). As shown in Fig. 8B, the BRET_max_ signal increased in Arf1-KO cells as expected as less AP-1 complex is activated whereas the BRET_50_ (Fig. 8C) was unaffected indicating that the association between the two subunits is normal in the absence of Arf1. Mammalian Arfs possess high levels of similarities in their primary sequences and are often localized in the same compartment suggesting they might have redundant functions. This is supported by studies showing that simultaneous depletion of multiple Arfs is required to observe certain phenotypes^31–33^. Our results suggest that Arf1 plays a specific role in the recruitment and activation of AP-1, though we cannot exclude that simultaneously knocking-out another Arf could lead to a more pronounced phenotype. We next performed rescue experiments by co-transfecting γ-RlucII and µ1-GFP10 with either Arf1^WT^-HA or Arf1^W172D^-HA in Arf1-KO cells. As shown in Fig. 8D, wild-type Arf1 brought the BRET_max_ signal to a level similar to HEK293T cells whereas Arf1^W172D^ only had a partial effect. This result confirms that even though Arf1^W172D^ interacts with AP-1 with a similar affinity as wild-type Arf1, it is less efficient in driving the conformational change of the complex. In order to determine if the observed conformational change is associated with activation and normal function of AP-1, we co-expressed Arf1-HA or Arf1^W172D^-HA with PSAP-myc in HEK293T or Arf1-KO cells and measured their ability to rescue the quantity of PSAP secreted outside the cells, a phenotype observed when disrupting normal trafficking pathways^34^. The overexpression of wild-type Arf1 was able to decrease the secretion of PSAP in Arf1-KO cells, whereas Arf1^W172D^ could not (Fig. 8E). These results show that, in addition to recruiting AP-1 to the membrane, Arf1 stimulates a conformational change necessary for the function of AP-1 *in vivo*.

### Membrane recruitment and activation by Arf1 increases AP-1 oligomerization

**Figure 9:**
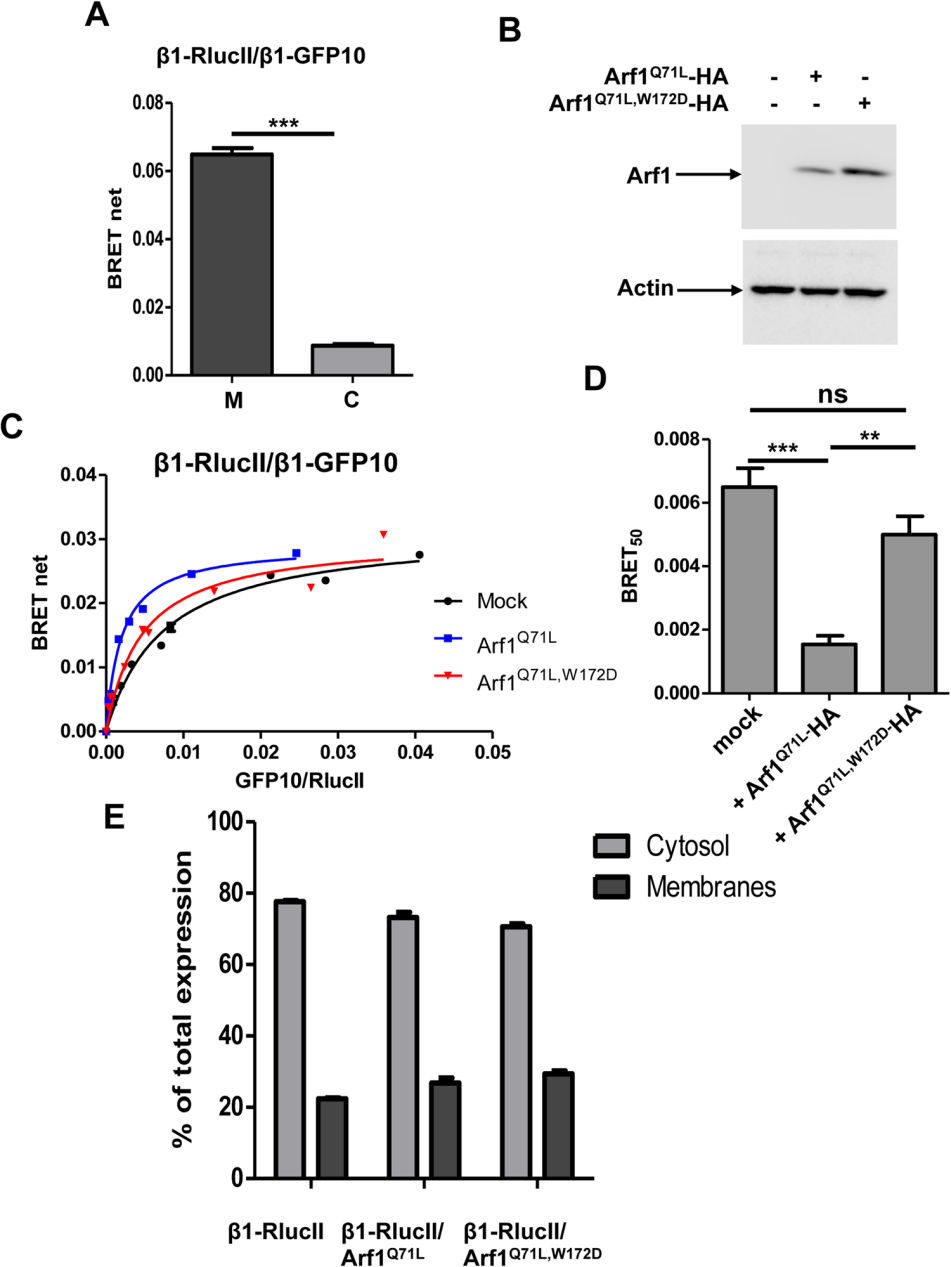
Arf1 increases AP-1 oligomerization in living cells. (**A**) HEK293T cells were transfected with β1-RlucII and β1-GFP10 and a membrane isolation assay was performed to separate cytosolic (**C**) and membrane fractions (M). BRET^2^ signals were measured in each fraction. BRET^2^ signals shown are observed at similar level of fluorescence/luminescence ratios. Data are shown as the mean ± SEM of three independent experiments analyzed by paired student *t*-test to assess statistical significance. ***P < 0.001. (**B**) The level of expression of Arf1^Q71L^-HA and Arf1^Q71L,W172D^-HA was determined by Western blot using an anti-HA antibody. (**C**) A low and constant amount of β1-RlucII and increasing concentrations of β1-GFP10 were co-transfected in HEK293T cells with or without Arf1^Q71L^-HA or Arf1^Q71L,W172D^-HA and BRET^2^ signals were measured. (**D**) BRET_50_ are displayed as mean ± SEM of 6 independent experiments and their statistical significance were assessed by ANOVA followed with Tukey’s post-hoc test. **P < 0.01; ***P < 0.001; ns, non significant. (**E**) A membrane isolation assay was performed on HEK293T cells transfected with β1-RlucII and Arf1^Q71L^-HA or Arf1^Q71L,W172D^-HA. The amount of β1-RlucII in cytosolic and membrane fractions was determined by measuring the luminescence coming from RlucII.

Previous experiments with purified adaptor proteins and synthetic liposomes showed that Arf1 can induce AP-1 oligomerization^30^ and models of AP-1 interaction with Arf1 suggest that AP-1 would need to bind Arf1 as a monomer first and then oligomerize at the membrane^12^. We sought to determine the monomeric/oligomeric assembly of AP-1 in the cytosol and membrane compartments. The cytosol and membrane compartments of HEK293T cells transfected with β1-RlucII and β1-GFP10 were separated by cell fractionation and BRET was measured in each fraction. As shown in Fig. 9A, the BRET signal coming from membrane compartments is significantly stronger than the BRET observed in the cytosol. The later may be explained by contamination of the cytosolic fraction with membranes or that a small proportion of AP-1 could exist as oligomer in the cytosol. We next performed BRET titration experiments in HEK293T cells expressing β1-RlucII or β1-GFP10 with Arf1^Q71L^-HA, Arf1^Q71L,W172D^-HA or mock transfected, to determine if recruitment of AP-1 to the membrane by Arf1 leads to its oligomerization (Fig. 9B and C). The BRET_50_ for the association between β1-RlucII and β1-GFP10 decreased by almost 4 fold in the presence of Arf1^Q71L^ (Fig. 9D) suggesting that recruitment of AP-1 by Arf1 increases its oligomerization. Interestingly, Arf1^Q71L,W172D^ is less efficient at driving AP-1 oligomerization (Fig. 9D), even though it is as or even more potent in increasing AP-1 membrane association than Arf1^Q71L^ (Fig. 9E). Together, these results indicate that higher level of AP-1 oligomerization requires both the membrane association and conformational changes associated with its activation.

## Discussion

The interaction of the multimeric adaptor protein (APs) complexes and COPI with Arf1, and their subsequent activation have been extensively studied *in vitro* ^12, 35, 36^. We took advantage of BRET to study the interaction between Arf1 and AP-1, and how this interaction leads to the activation of the complex in live cells using full-length proteins in their native environment.

The crystal structure of Arf1 interacting with AP-1 revealed multiple interaction interfaces. The switch I/II regions of Arf1 mediates interaction interfaces with both β1 and γ subunits of AP-1. Based on structural data, at least 3 residues in β1 (Gln59, Ile85 and Asn89) should be important for binding Arf1^12^. By overlaying β1 and γ, a model for Arf1 bound to γ was generated and identified potentially important residues (Leu68, Leu71 and Leu102). *In vitro* binding experiments using mutations in both β1 and γ prevented a stable interaction with Arf1. Our *in vivo* data using BRET supports this conclusion. We also tested β1^Q59E^, which was not reported in the *in vitro* experiments. We found no changes in the interaction between Arf1 and AP-1 suggesting a lesser contribution of this residue to the AP-1/Arf1 interaction *in vivo*.

Based on the crystal structure, several residues in the switch I/II domains of Arf1 were predicted to be important for its interaction with AP-1. *In vitro* studies showed that Arf1 switch I residues Ile49 and Val53 and switch II residues Lys73, Leu77 and His80 are required for its interaction with AP-1. However, other switch I residues (Phe51 and Asn52) and switch II residues (Ile74 and Tyr81) were also predicted to mediate this interaction but their roles in the interaction were not reported. Our *in vivo* studies confirmed the role of Ile49 in binding AP-1. Interestingly, mutations at Val53, Leu77 and His80 did not affect binding between Arf1 and AP-1 using BRET. Furthermore, when we tested Phe51, Asn52, Ile74 and Tyr81, all had significant effects on the interaction. Our *in vivo* experiments confirm the role of switch I/II in mediating the interaction between Arf1 and AP-1. However, the discrepancy observed between the *in vitro* and *in vivo* experiments highlights the possibility that the interface conformation observed *in vitro* is slightly different. This could be due to using truncated proteins (Arf1, β1 and/or γ), the lack of a membrane environment or the lack of post-translational modifications in the *in vitro* experiments.

A third interface predicted by the crystal structure between the backside of Arf1 and γ was tested using various Arf1 mutations. It was predicted that Ala136, Ala137 and Trp172 of Arf1 were important in the binding to γ. *In vitro*, these mutations (Arf1^A136,137H^ and Arf1^W172D^) did not prevent the interaction between Arf1 and AP-1, but surprisingly increased it. These residues were suggested to be involved in the activation of the complex based on an *in vitro* assay where Arf1^W172D^ is less efficient in promoting cargo protein binding to AP-1. In our *in vivo* studies, we found no significant changes in the affinity between these Arf1 mutants and AP-1, confirming that they were not necessary for binding. By tagging the γ and µ1 subunits to RlucII and GFP10 respectively, we were able to directly detect the conformational changes associated with the unlocking of AP-1 in live cells and confirm the allosteric function of Trp172 in Arf1 in the activation of AP-1. Moreover, using an Arf1-KO cell line displaying aberrant secretion of the lysosomal protein prosaposin, we were able to correlate the unlocking of AP-1 to its function in lysosomal trafficking, as Arf1^W172D^ was less efficient than Arf1^WT^ in rescuing prosaposin secretion.

Although the crystal structure of Arf1-GTP with AP-1 revealed a dimeric 2:2 Arf1: AP-1 assembly, modeling suggests that the γ/switch I/II recruitment interface is incompatible with binding of a dimer of AP-1^12^. Therefore, it was proposed that Arf1 first binds AP-1 in its closed state as a monomer and once on the membrane, AP-1 would dimerize. Consistent with this hypothesis, we found that AP-1 exists predominantly as a monomer in the cytosol whereas it forms dimers or oligomers when recruited to membranes. Furthermore, by overexpressing a constitutively active form of Arf1 (Arf1^Q71L^) we were able to increase the oligomerization of AP-1 whereas the activation deficient mutated form of Arf1 (Arf1^Q71L,W172D^) resulted in less oligomerization. This suggests that higher order oligomerization of AP-1 requires the conformational changes associated with the activation of the complex. Interestingly, a recent report on the complex between AP-1, Arf1 and the HIV Nef protein suggest that Arf1^W172D^ is less efficient in driving higher order oligomers of AP-1 based on size exclusion chromatography^35^.

In summary, we have used BRET to determine the interaction sites between Arf1 and AP-1, as well as to directly study the conformational changes in AP-1 driven by Arf1 in live cells. As far as we know, this is the first time that conformational changes in AP-1 have been directly monitored in live cells. This technique could be efficiently used to study other protein complexes *in vivo* including the association between small GTPases and their effectors. We propose that BRET is a useful tool to study protein complexes in live cells because full-length proteins are expressed in their native environments that are post-translationally modified. BRET has the additional benefit of being significantly less labor intensive than purifying proteins from bacterial lysates and can be performed in 96 or 384-well plates which makes its suitable for high-throughput screening.

## Materials and Methods

### Reagents and antibodies

All reagents, unless specified, were purchased from Fisher Scientific (Ottawa, ON). Dulbecco’s modified Eagle’s medium, fetal bovine serum and penicillin-streptomycin were purchased from Wisent (St-Bruno, Qc, Canada). Linear 25 kDa polyethyleneimine (PEI) was from Polysciences Inc. (Warrington, PA). Coelenterazine 400a and coelenterazine h were purchased from Prolume Ltd. (Pinetop, AZ). Brefeldin A was purchased from Molecular Probes (Eugene, OR). The protease inhibitor cocktail was purchased from Sigma-Aldrich (Oakville, ON). The following mouse monoclonal antibodies were used: anti-hemagglutinin (anti-HA) antibody (MMS-101P, Cedarlane Laboratories, Burlington, ON); anti-myc antibody (MMS-105P, Cedarlane Laboratories); anti-γ antibody (A4200, Sigma-Aldrich, Oakville, ON); anti-actin (612657, BD Biosciences, Mississauga, ON); anti-*Renilla* luciferase (MAB4410) antibody (16932-1-AP) was purchased from EMD Millipore (Billerica, MA). The following rabbit polyclonal antibodies were used: anti-GFP antibody (ab290, Abcam, Cambridge, MA); anti-ARF1 antibody (20226-1-AP, Proteintech, Chicago, IL). Anti-mouse and anti-rabbit horseradish peroxidase-conjugated IgG were from GE Healthcare (Chalfont St.Giles, Buckinghamshire, UK).

### Eukaryotic expression vectors

Human Arf1 was amplified by PCR minus the methionine initiation site and subcloned in-frame upstream of RlucII into the pcDNA 3.1 (+) vector to create the Arf1-RlucII construct. Human γ, β1 and µ1 were amplified by PCR minus the stop codon and subcloned in-frame 5′ of RlucII into the pcDNA 3.1 Hygro (+) vector to create γ-RlucII, β-RlucII and µ1-RlucII. Human γ, β1 and µ1 were amplified by PCR minus the stop codon and subcloned in-frame 5′ of GFP10 into the pcDNA 3.1 (+) vector to create γ-GFP10, β-GFP10 and µ1-GFP10. PSAP-myc^37^ and Arf1-HA^38^ were previously described. The various mutants were engineered by site-directed mutagenesis from the previously described constructs. The various mutations introduced in to Arf1 (Figure S1), β1 (Figure S2A and B) or γ (Figure S2C) did not alter their expression compared to wild-type versions of the proteins.

### Cell culture and transient transfections

Human embryonic kidney 293 cells (HEK293T) were cultured in Dulbecco’s modified Eagle’s medium (DMEM) containing L-glutamine supplemented with 10% fetal bovine serum and 5% penicillin/streptomycin at 37 °C in a humidified chamber at 95% air and 5% CO_2_. The cells were seeded at a density of 2 × 10^5^/well for 12 well plates, 5 × 10^5^/well for 6 well plates or 3 × 10^6^ for 10 cm dishes, 24 hours prior to transfection. 1 µg/well for 12 well plates, 2 µg/well for 6 well plates or 8 µg for 10 cm dishes of total DNA were used for transient transfection using linear 25 kDa polyethyleneimine (3 µg PEI/µg DNA).

### BRET titration experiments

The various vectors for proteins fused to RlucII were co-transfected in HEK293T cells seeded in 12-well plates at a low and constant quantity (6 ng for Ar1-RlucII constructs, 40 ng for β1-RlucII and 70 ng for γ-RlucII) with increasing quantities of the vectors for the different proteins fused to GFP10 (from 10 ng up to 600 ng). Approximately 48 hours later, the cells were washed, detached in PBS and transferred to white opaque 96-well plates (Greiner) at a density of ~100 000 cells. Total fluorescence was first measured with the infinite M1000 Pro plate reader from Tecan Group Ltd. (Mannedorf, Switzerland) with the excitation and emission set at 400 nm and 510 nm respectively. The BRET^2^ substrate coelenterazine 400a was then added to all wells (5 µM final concentration) and the BRET^2^ signal was measured 3 min later on the infinite M1000 Pro. The BRET^2^ signal was calculated as a ratio of the light emitted at 525 ± 15 nm over the light emitted at 410 ± 40 nm. The BRETnet signal was calculated as the difference between the BRET^2^ signal in cells expressing both GFP10 and RlucII constructs and the BRET^2^ signal from cells where only the RlucII fused construct was expressed. BRET titration curves were generated by expressing comparable levels of wild-type and mutant versions of the various RlucII (Figure S1) and GFP10 constructs (Figure S2). To ensure that increasing plasmid levels resulted in an increased expression of the fluorescence signal per cell, and not the increase in the number of cells expressing a similar level of fluorescence, we transfected HEK293T cells in 6 well plates with various concentrations of β1-GFP10 (50, 100, 250 and 1000 ng), and determined the average fluorescence intensity per cell using immunofluorescence microscopy and image analysis (Figure S7).

### BRET measurements for Brefeldin A treatment

Appropriate quantities of γ-RlucII(70 ng)/µ1-GFP10 (150–200 ng) or β1-RlucII (40ng)/β1GFP10 (300 ng) were cotransfected in HEK293T cells to obtain a BRET_max_ for each pair of proteins. 24 hours after transfection, the cells were detached in DMEM and transferred to sterile white opaque 96-well plates. 24 hours later, the cells were washed with DMEM without phenol red and treated with Brefeldin A (10 µM) or vehicle in DMEM without phenol red for 15 min at 37 °C. The total fluorescence and BRET^2^ signals were measured as described above.

### BRET rescue experiments

Various quantities of plasmid coding for γ-RlucII (70 to 100 ng) and µ1-GFP10 (75 to 200 ng) were cotransfected in HEK293T WT or Arf1-KO cells with or without a low quantity (100 ηg) of a plasmid coding for Arf1-HA or Arf1W172D-HA. Approximately 48 hours after transfection, cells were washed and detached in PBS then seeded at a density of approximately 100 000 cells/well in white opaque 96-well plates. The total fluorescence and BRET^2^ were measured as described above. Conditions of similar luminescence and fluorescence levels were used for analysis.

### Membrane isolation assay

This assay was previously described^39^. Briefly, approximately 48 hours after transfection, HEK293T cells were washed with PBS, detached with 5 mM EDTA in PBS and following centrifugation, the pellet was snap frozen with liquid nitrogen. The samples were then resuspended in buffer 1 (0.1 M Mes-NaOH pH 6.5, 1 mM MgAc, 0.5 mM EGTA, 200 µM sodium orthovanadate, 0.2 M sucrose) and centrifuged at 10 000 g for 5 min at 4 °C. The supernatants (cytosolic fraction) were collected and the pellets (membrane fraction) were resuspended in an equal volume of buffer 1. 40 µl of each fraction were transferred to a white 96 well plate (Greiner) and coelenterazine h was added to a 5 µM final concentration. Total luminescence was measured in the Infinite M1000 Pro plate reader (Tecan, Morrisville, NC).

### Western blotting

Approximately 48 hours after transfection with the indicated plasmids, HEK293T cells seeded in 6 or 12 well plates were washed with PBS, detached with 5 mM EDTA in PBS and lysed in TNE buffer (150 mM NaCl, 10 mM Tris-HCl pH 7.5, 5 mM EDTA, 0.5% Triton X-100, 0.5%) containing protease inhibitors for 1 hour at 4 °C under gentle agitation. Lysates were clarified by centrifugation at 13 000 rpm for 15 minutes at 4 °C and 4X SDS-PAGE loading buffer was added to the supernatants to a 1X final concentration (50 mM Tris-HCL pH 6.8, 2% SDS, 01% bromophenol blue, 10% glycerol, 1.25% β-mercapthoethanol). Proteins were then resolved on SDS-PAGE, transferred to nitrocellulose membranes and detected by immunoblotting using the indicated antibody.

### Secretion assay

6 well plates seeded with WT or KO HEK293T cells were transfected with PSAP-myc and the indicated plasmids. Approximately 48 hours after transfection, cells were washed once with PBS and incubated with 1 ml of Opti-Mem for 5 hours at 37 °C in a humidified chamber at 95% air and 5% CO_2_. The Opti-Mem containing secreted proteins was collected and trichloroacetic acid was added to a final concentration of 20% to precipitate proteins. After a 1 hour incubation at 4 °C, samples were centrifuged for 5 min at 21 000 g at 4 °C. The pellets were washed with 200 µl of cold acetone, centrifuged and washed again with acetone. After evaporating the acetone at 95 °C for 1 min, the pellets were resuspended in 2X SDS-PAGE loading buffer. In parallel, total cell lysates were prepared as described before and proteins were then resolved on SDS-PAGE, transferred to nitrocellulose membranes and detected by immunoblotting using the indicated antibody.

### Co-immunoprecipitation

HEK293T cells seeded in 10 cm dishes were transfected with the indicated plasmid and 48 hours later, the cells were washed with PBS, detached with PBS/EDTA and lysed in TNE buffer for 2 hours at 4 °C under gentle agitation. The lysates were clarified by centrifugation at 13 000 rpm for 25 min at 4 °C and then pre-cleared with protein-G sepharose for 1 hour at 4 °C. Protein-G sepharose and the indicated antibodies were added to the supernatants followed by an overnight incubation at 4 °C. The precipitates were washed five times in TNE buffer and the proteins eluted 1 h at room temperature in 50 µl of SDS-PAGE loading buffer (125 mMTris-HCl, pH 6.5, 4% SDS, 2 M urea, 5% glycerol, 0.1% bromophenol blue). Proteins were then resolved on SDS-PAGE, transferred to nitrocellulose membranes and detected by immunoblotting using the indicated antibody.

### Immunofluorescence microscopy

HEK293T cells seeded on 12mm coverslips were transfected with the indicated plasmid and 36 later, the cells were washed with PBS and fixed in 4% paraformaldehyde for 15 minutes. The cells were then washed with PBS, and incubated with 0.1% saponin for 10 minutes. The cells were subsequently immunostained with primary antibody for 1 hours at RT, washed in PBS and the incubated with the appropriate secondary antibody. Cells were then incubated with DAPI for 1 minute, prior to being mounted onto glass slides with Fluoromount G and sealed with nail polish. Images were acquired on a Zeiss inverted microscope. Image analysis to quantify fluorescence intensity was performed using Zen Pro software (Carl Zeiss Inc, Mississauga, ON).

### Data and statistical analysis

All graphs were generated and analyzed using GraphPad Prism (GraphPad Software Inc.) and the BRET_50_ and BRET_max_ were extrapolated from non-linear regression fits using the one-site saturation binding fitting. All the titration curves shown are representative of at least three independent experiments. Statistical analysis were done using student’s *t*-test or one-way analysis of variance (ANOVA) followed by Tukey’s or Dunnet’s post-hoc tests as appropriate (see figure legends).

## Electronic supplementary material

Supplemental Information
