# Supplementary material for: In vivo monitoring of the recruitment and activation of AP-1 by Arf1
Source: Sci Rep. 2017 Aug 2;7:7148. doi: 10.1038/s41598-017-07493-1 (PMC5540999; doi:10.1038/s41598-017-07493-1)
Supplement: Supplementary file 1 — Supplemental Information [file 41598_2017_7493_MOESM1_ESM.pdf]

Supplemental Information

In vivo monitoring of the recruitment and activation of AP-1 by Arf1

Etienne Sauvageau [a], Peter J. McCormick [b] and Stephane Lefrancois [a,c,*]
[a] Centre INRS-Institut Armand-Frappier, INRS, Laval, Canada H7V 1B7
[b] Faculty of Health and Medical Sciences, School of Veterinary Medicine, University of Surrey, Guildford, UK GU27XH
[c] Department of Anatomy and Cell Biology, McGill University, Montreal, Canada H3A 2B2

* To whom correspondence should be addressed: Stephane Lefrancois, Centre INRS-Institut Armand-Frappier, INRS, Laval, Canada H7V 1B7 Tel.: 450-687-5010 ext. 8860; Fax: 450 686-5501; E-mail: stephane.lefrancois@iaf.inrs.ca

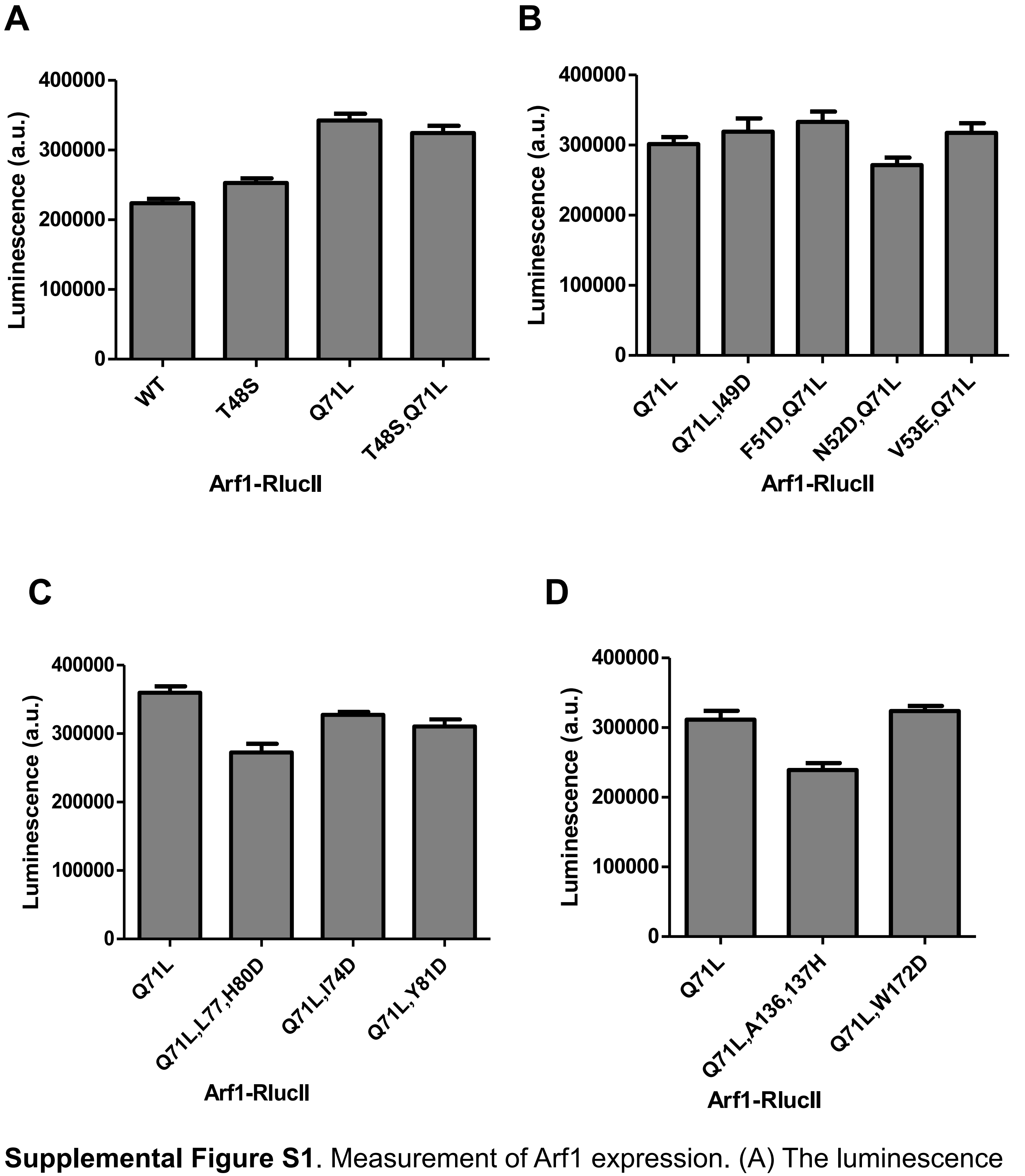

Supplemental Figure S1. Measurement of Arf1 expression. (A) The luminescence Emission at 410 nm from the BRET titration experiment from Figure 2A was measured to determine The expression of each Arf1 construct to ensure comparable levels of expression. (B) Same as in A, but for the BRET titration curve in Figure 4A. (C) Same as in A, but for the BRET titration curve in Figure 4C. (D) Same as in A, but for the BRET titration curve in Figure 6A.

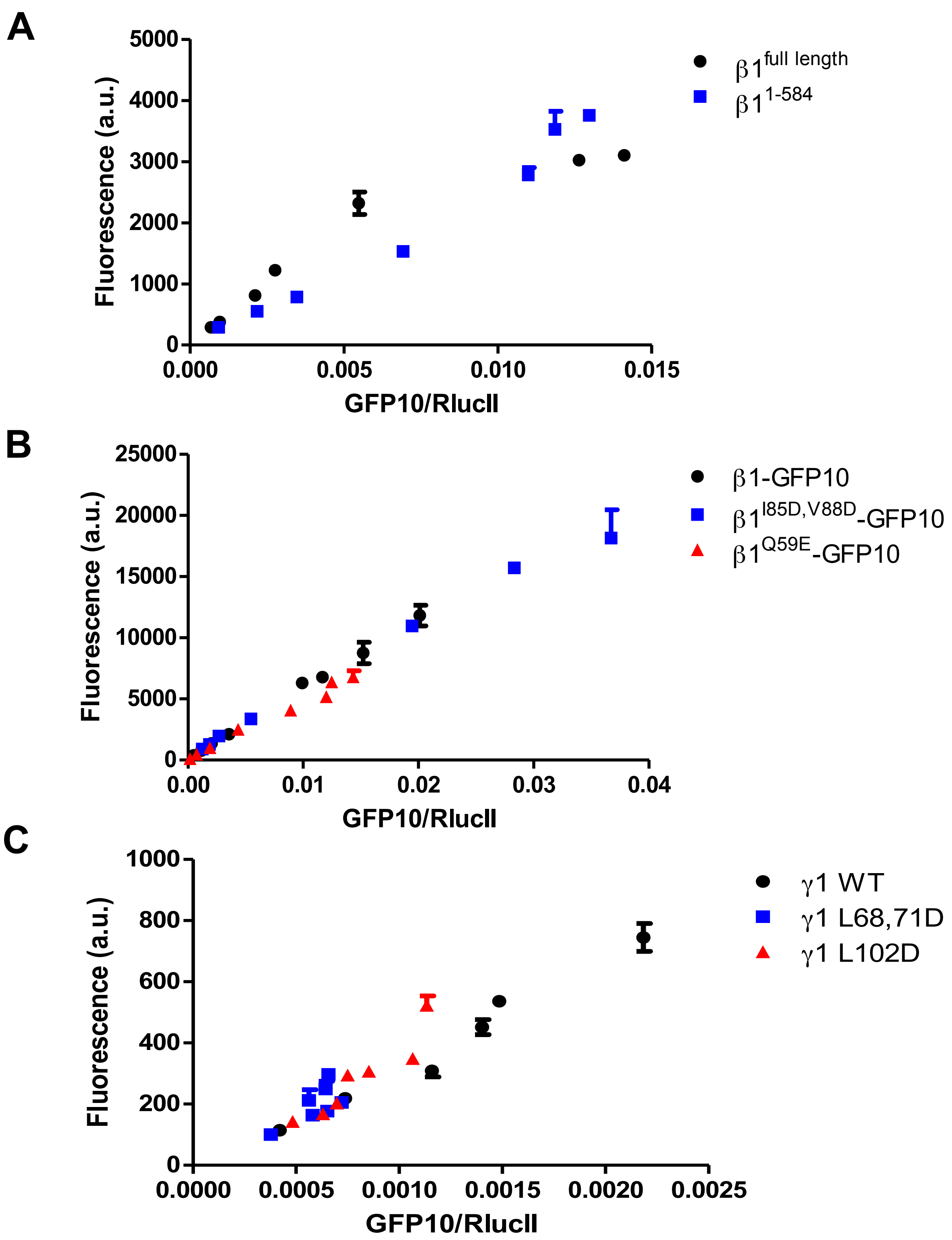

Supplemental Figure S2. Determining the expression of acceptor proteins. (A) In order to compare the expression levels of wild-type β1-GFP10 versus the truncated form lacking its C-terminal appendage ($\beta1^{1\text{-}584}$), increasing amount of DNA was transfected for each plasmid and the fluorescence emission measured. (B) The same experiment as in A was performed, but with wild-type and mutant versions of β1 as indicated. (C) The same experiment as in A was performed, but with wild-type and mutant versions of γ-GFP10 as indicated.

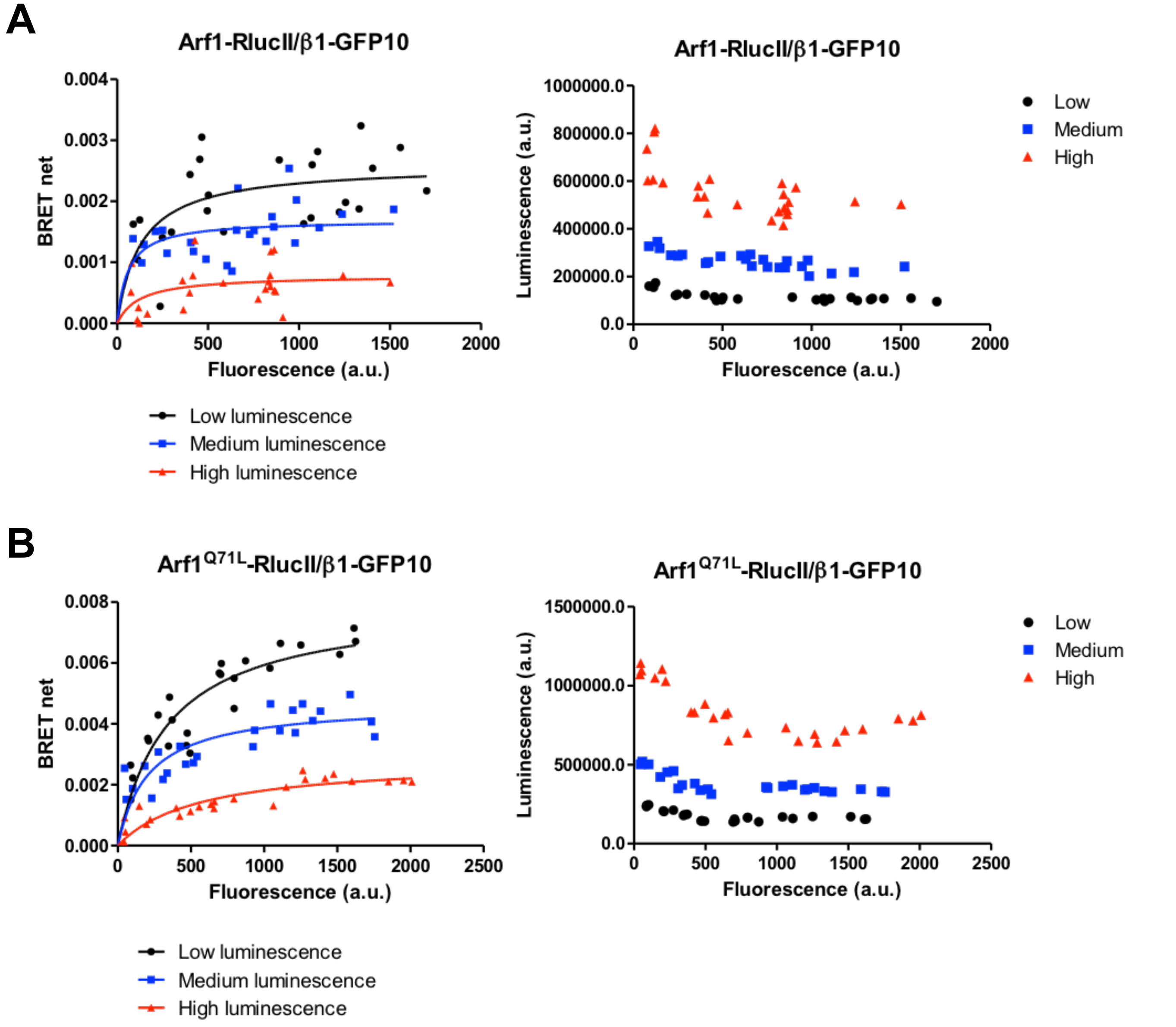

Figure S3. BRET titration curves at different levels of donor expression. (A) A Low (black curve), medium (blue curve) or high (red curve) quantity of Arf1-RlucII Were co-transfected along with increasing concentrations of β1-GFP10 in HEK293T cells. The fluorescence and $BRET^2$ were measured in parallel and $BRET_{net}$ levels are plotted as a function of fluorescence. The results shown are representative of at least three independent experiments. The lower graph displays the level of luminescence for low (113210 a.u.), medium (266647 a.u.) or high (557741 a.u.) quantity of Arf1-RlucII transfected. (B) Same as in A, but cells were co-tranfected with $Arf1^{Q71L}$-RlucII. The lower graph displays the level of luminescence for low (171653 a.u.), medium (378993 a.u.) or high (834063 a.u.) quantity of $Arf1^{Q71L}$-RlucII transfected.

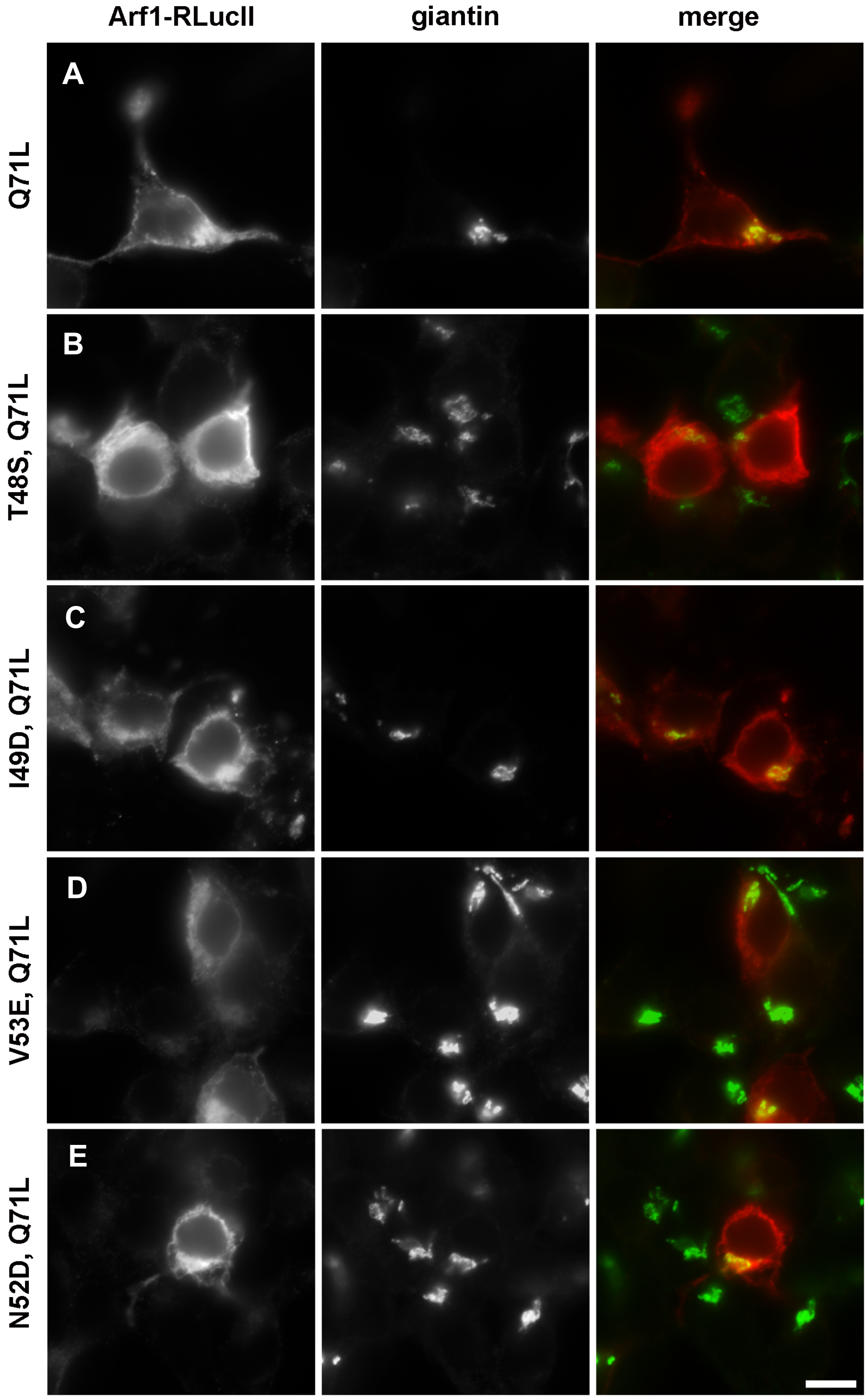

Supplemental Figure S4. The various mutant Arf1 constructs still co-localize with giantin. (A) HEK293T cells were transfected with Arf1^{Q71L}-RlucII, fixed in 4% paraformaldehyde and immunostained with anti-RLuc and anti-giantin antibodies. (B) Same as in A, but transfected with Arf1^{T48S,Q71L}-RlucII. (C) Same as in A, but transfected with Arf1^{I49D,Q71L}-RlucII. (D) Same as in A, but transfected with Arf1^{V53E,Q71L}-RlucII. (E) Same as in A, but transfected with Arf1^{N52D,Q71L}-RlucII. Scale bar = 10 mm.

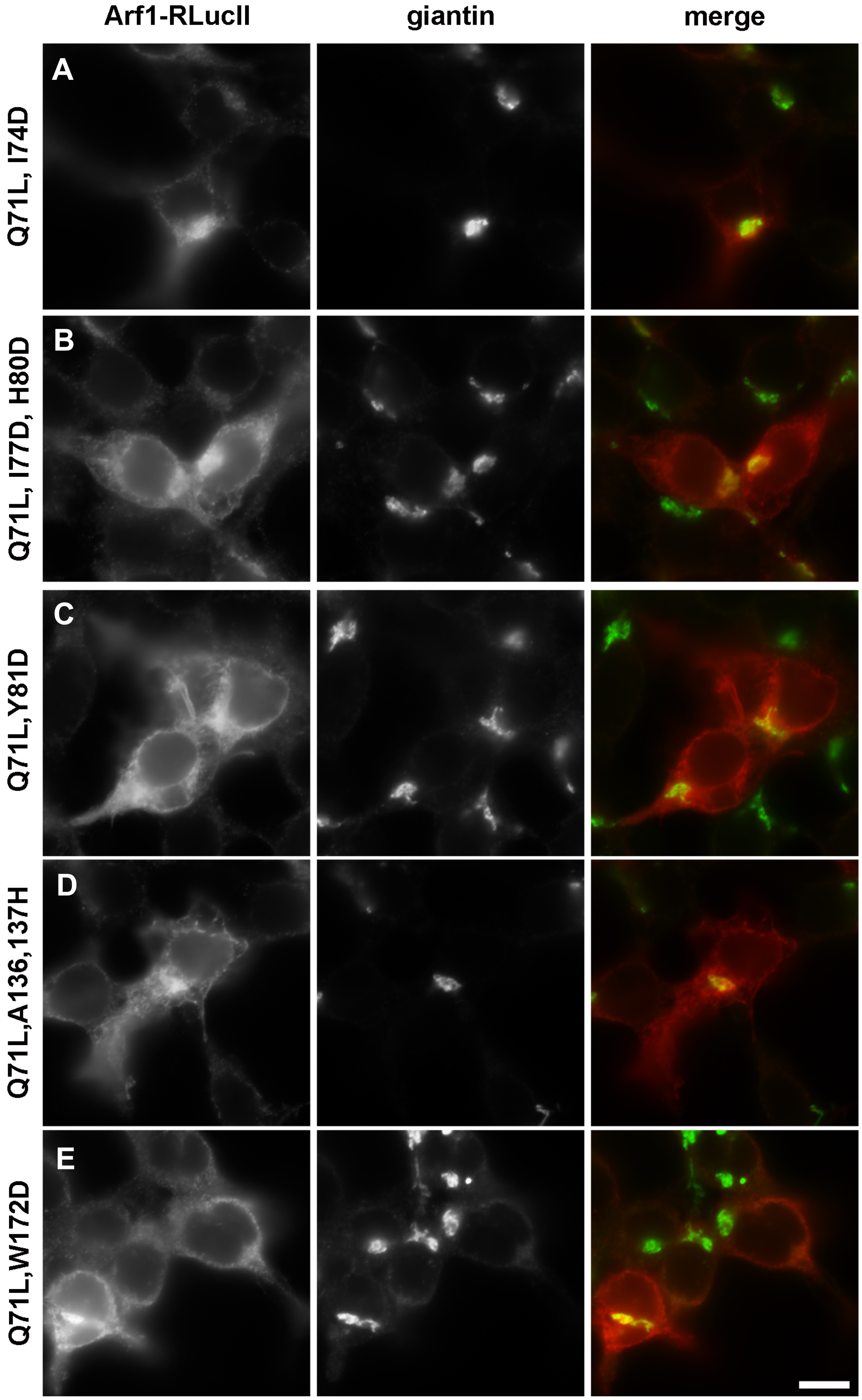

Supplemental Figure S5. The various mutant Arf1 constructs still co-localize with giantin. (A) HEK293T cells were transfected wild-type Arf1^{Q71L,I74D}-RlucII, fixed in 4% paraformaldehyde and immunostained with anti-RLuc and anti-Giantin anti-bodies. (B) Same as in A, but transfected with Arf1^{Q71L,I77D,H80D}-RlucII. (C) Same as in A, but transfected with Arf1^{Q71L,Y81D}-RlucII. (D) Same as in A, but transfected with Arf1^{Q71L,A136,137H}-RlucII. (E) Same as in A, but transfected with Arf1^{Q71L,W172D}-RlucII. Scale bar = 10 mm.

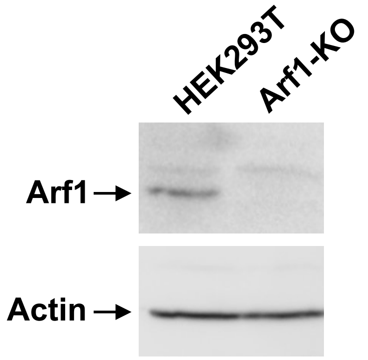

Supplemental Figure S6. An Arf1 knockout (Arf1-KO) cell line was engineered using CRISPR/Cas9. HEK293T and Arf1-KO cells were lysed in TNE buffer and endogenous proteins were detected by Western blot using antibodies against Arf1. Western blotting against actin was used as a loading control. Western blot images were cropped for space considerations.

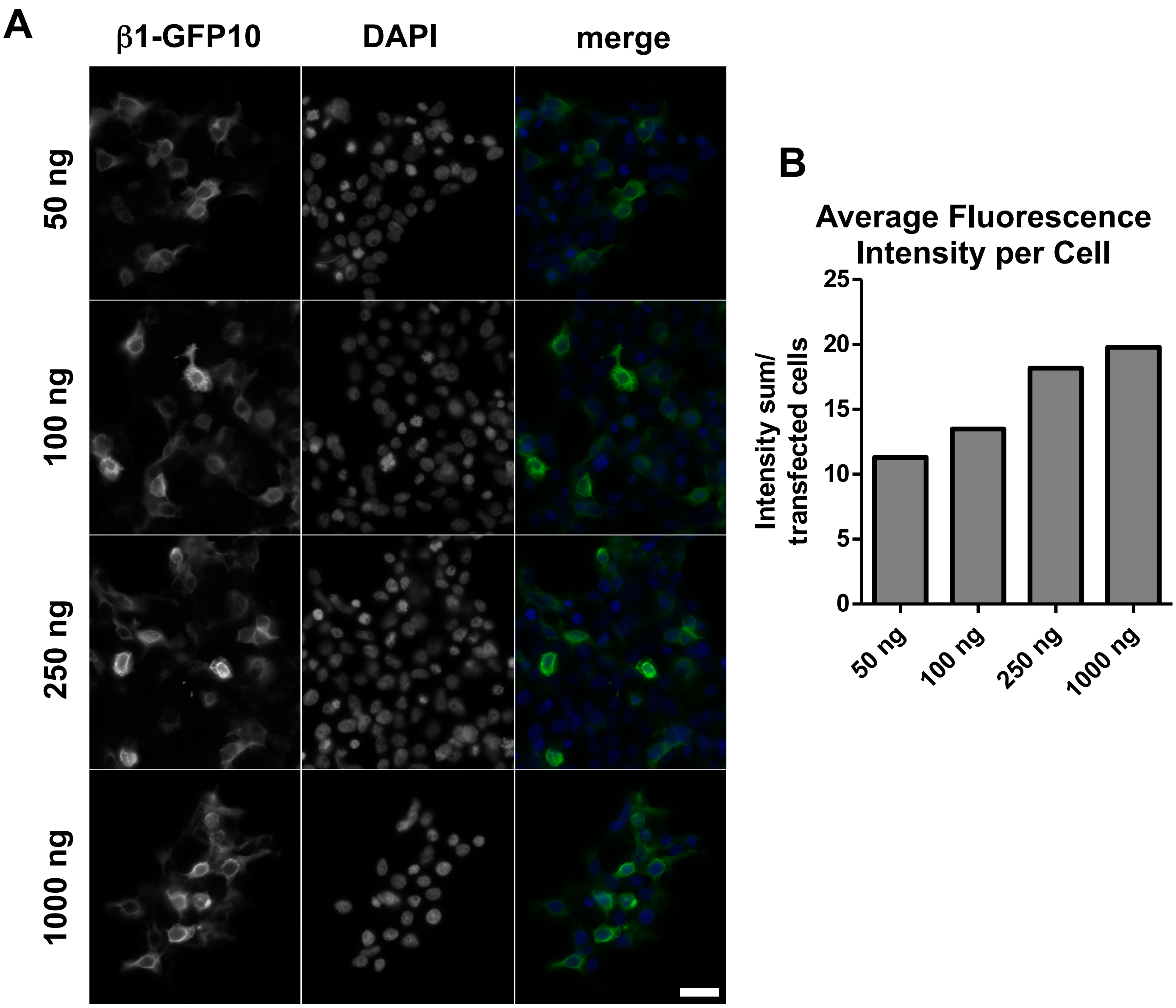

Supplemental Figure S7. Increasing the amount of DNA results in increased fluorescence intensity per cell. (A) HEK293T cells were tranfected with increasing amounts of plasmid DNA to express b1-GFP10. 36 hours post-transfection, cells were fixed in 4% paraformaldehyde and immunostained with anti-GFP antibody and DAPI. Scale bar = 30 mm. (B) The mean fluorescence intensity per cell was plotted per transfection condition.

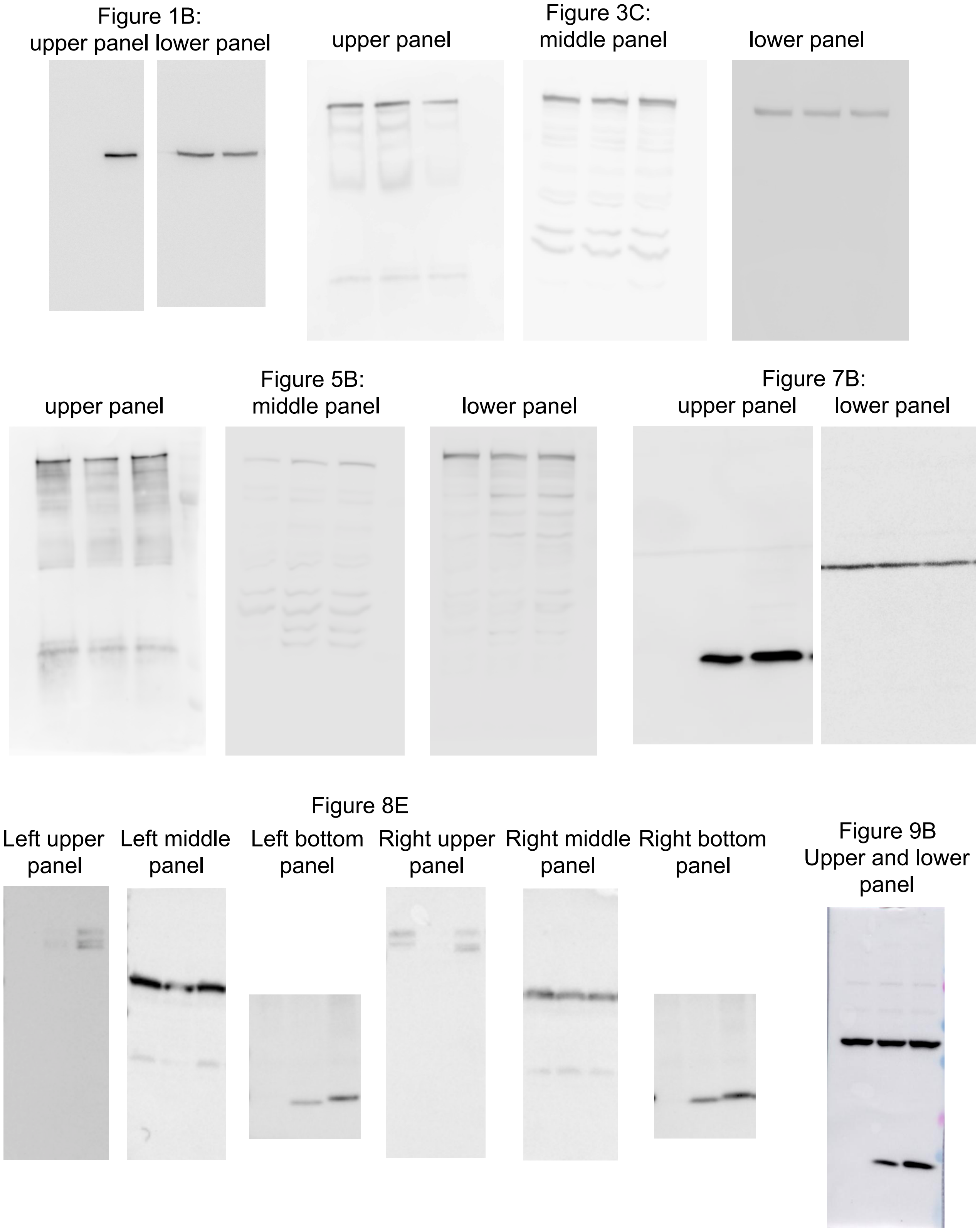

Supplemental Figure S8: Source data for Western blots used in figure 1, 3, 5, 7, 8 and 9 as identified above
